# Insights from the comparison of genomic variants from two influenza B viruses grown in the presence of human antibodies in cell culture

**DOI:** 10.1371/journal.pone.0239015

**Published:** 2020-09-14

**Authors:** Ewan P. Plant, Hasmik Manukyan, Majid Laassri, Zhiping Ye

**Affiliations:** Office of Vaccine Research and Review, US Food and Drug Administration, Silver Spring, Maryland, United States of America; University of South Dakota, UNITED STATES

## Abstract

Understanding the extent and limitation of viral genome evolution can provide insight about potential drug and vaccine targets. Influenza B Viruses (IBVs) infect humans in a seasonal manner and causes significant morbidity and mortality. IBVs are negative-sense single-stranded RNA viruses with a segmented genome and can be divided into two antigenically distinct lineages. The two lineages have been circulating and further evolving for almost four decades. The immune response to IBV infection can lead to antibodies that target the strain causing the infection. Some antibodies are cross-reactive and are able to bind strains from both lineages but, because of antigenic drift and immunodominance, both lineages continue to evolve and challenge human health. Here we investigate changes in the genomes of an IBVs from each lineage after passage in tissue culture in the presence of human sera containing polyclonal antibodies directed toward antigenically and temporally distinct viruses. Our previous analysis of the fourth segment, which encodes the major surface protein HA, revealed a pattern of change in which signature sequences from one lineage mutated to the signature sequences of the other lineage. Here we analyze genes from the other genomic segments and observe that most of the quasispecies’ heterogeneity occurs at the same loci in each lineage. The nature of the variants at these loci are investigated and possible reasons for this pattern are discussed. This work expands our understanding of the extent and limitations of genomic change in IBV.

## Introduction

An understanding of the genomes of pathogens can provide a useful context for the prevention of disease. Genomes evolve over time. They are shaped by external evolutionary pressures and constrained by internal regulatory and protein coding requirements.

Influenza B viruses (IBVs) are negative strand segmented viruses and belong to the Orthomyxoviridae family. The fundamental nature of influenza genomes has been described many years ago and continued research has further refined our understanding [1]. There are some differences in the genome of IBV compared to the better studied influenza A viruses. Both viruses have eight genomic segments but the number of known open reading frames (ORFs) differs. The major ORFs on each segment are analogous between influenza A and IBV genomes [2]. The three largest segments encode the polymerase subunits PB2, PB1 and PA, required for both transcription and replication [3]. The fourth and sixth segments encode the glycoproteins hemagglutinin (HA) and neuraminidase (NA). The fifth segment encodes the nucleoprotein [4]. The seventh segment has two ORFs encoding matrix proteins [5]. The eighth segment encodes two nonstructural proteins [6]. An additional ORF named NB was identified in the NA segment of IBV [7]. The mechanisms behind the translation of the various ORFs differ among influenza viruses, for example, the second ORF in the M segment from influenza A is translated from an mRNA splice variant but the second ORF in IBV is translated by a termination-reinitiation event [8, 9]. Several overlapping ORFs and mRNA splice variants have recently been identified in influenza A viruses [10, 11] and deep sequencing (NGS) of mRNA from IBV infected cells has also identified splice variants [12]. The overlapping genes in viruses can evolve asymmetrically with each encoded protein subject to different selection pressures [13].

IBVs can be divided into two antigenically distinct lineages [14]. These two lineages have been circulating and further evolving for almost four decades. Strains isolated prior to 1980 were genetically and serologically related [15]. The major surface proteins, HA and NA, from influenza A have been shown to work in concert [16, 17]. It was also shown that the fourth and sixth genomic segments mutate in a coordinated manner [18]. More recent knowledge of epistatic interactions in influenza A is available due to the capacity of saturation mutagenesis and deep sequencing [19]. Similar work has not yet been done for IBV.

Replication of the viral genome is an imperfect process, and this is reflected in the diversity of IBV sequences in everything from clinical isolates to genetically engineered strains. Genomic mutations frequently arise due to low polymerase fidelity [20]. Viral quasispecies are present in both natural and cultivated isolates. A small study by Rutvisuttinunt et al., revealed that clinical influenza isolates had more nucleotide diversity than the cultured isolates [21]. Wasik et al., demonstrated that viruses derived from plasmids also presented as a quasispecies with low levels of variants that didn’t become fixed [22]. Both antigenic and non-antigenic changes that become fixed can affect viral fitness [23]. Some deleterious mutations (that alter receptor binding avidity, enzyme kinetics, or protein stability for example) may be compensated by epistatic mutations [17]. The loss in viral fitness or mutational load can also reduce the probability that a variant becomes fixed in the population of an infected host [24]. Our understanding of these processes and genome stability can help fuel vaccine and antiviral drug design.

Antibodies are known to provide protection against disease and vaccination is a cost-effective approach to protect the population from influenza [25]. Human IgGs that target the IBV HA have been described [26–30]. The mechanism of action can include direct neutralization of viral infectivity and the engagement of host effector cells [31]. Cross-reactive antibodies generated against IBVs have been shown to be protective in vitro [32]. In addition, the differences in antibody responses toward the major antigenic sites of IBV in mice, ferrets and humans have been described [33].

Traditional influenza vaccines are designed so that antigens from the major surface proteins, HA and NA, are presented to the host and the subsequent immune response provides some level of protection [34]. However, antigenically distinct influenza viruses continue to cause disease in the human population. The variable effectiveness of seasonal influenza vaccines, and the pandemic potential due to some influenza viruses jumping from one species to another, has fueled the push for universal influenza vaccines that can provide protection against a broader range of viruses [35–37]. In addition to expanding the range of current vaccines, new vaccines using novel approaches are being developed. Bioinformatic tools are being used to identify epitopes which can be developed into vaccines for influenza A [38, 39]. Epitopes for M2 and NP proteins been identified as vaccine candidates and are being developed [37, 40].

Here we analyze IBV genomes to better understand how they evolve. We expand on our previous analyses of influenza B viruses passaged in tissue culture in the presence of human monoclonal antibodies. The sera were from subjects vaccinated with IBV strains that are antigenically distinct from the passaged viruses. Prior analyses revealed some sera-specific changes in which identical mutations arose in escape viruses from both lineages that altered antigenicity [41]. In addition the analyses also revealed that some changes in the HA segment made the virus from one lineage appear more like the other lineage [41]. Many of these changes were outside known antigenic sites. We postulated that such changes were limited to specific genomic loci and that genomic constraints may play a role. If true, mutations would also be constrained to specific loci in other parts of the genome not associated with antigenicity for viruses from both lineages. The current work analyzes variants present in the other segments, including those that haven’t become fixed. We provide an overview of the loci among the genomes that in which variants in the quasispecies change in frequency.

## Methods

The methods for virus growth, library preparation and Illumina sequencing have been described elsewhere [41]. Briefly, one Yamagata-lineage (B/Phuket/3073/2013) and one Victoria-lineage (B/Colorado/06/2017) IBV were propagated in tissue culture in the presence of sera from vaccine recipients. The viruses were passaged 1–4 times in MDCK cells [42] with sera from subjects who received trivalent inactivated influenza vaccines containing either the 2003 or the 2011 Victoria-lineage IBV antigens [43]. Six different sera were used resulting in six B/Phuket/3073/2013 viruses and six B/Colorado/06/2017 escape viruses in addition to the parental viruses passaged once without sera (Table 1). The initial virus titers used for passage were 4 and 32 for B/Phuket/3073 and B/Colorado/06/2017 respectively. Higher titers were used for the Victoria-lineage virus because sera from subjects vaccinated with Victoria-lineage antigens was expected to contain higher titers of antibodies targeting Victoria-lineage IBVs. Different dilutions (1:10, 1:40 and 1:160) of serum were used and virus obtained using the least dilute serum samples was used for subsequent passages. RNA was extracted using the QIAamp Viral RNA Mini Kit (Qiagen, Germantown, MD) following the manufacturer’s instructions. The RNA library was prepared using NEB Next Ultra RNA Library Prep Kit for Illumina (New England BioLabs, Ipswich, MA). The DNA fragments were ligated to Illumina paired end adaptors, amplified and purified using magnetic beads (Agencourt AMPure PCR purification system, Beckman Coulter, Brea, CA). Deep sequencing was performed using MiSeq (Illumina, San Diego, CA). The raw sequencing reads were analyzed using the High-performance Integrated Virtual Environment [HIVE: [44, 45]]. Raw sequence data is available at https://www.ncbi.nlm.nih.gov/bioproject/PRJNA655757/.

**Table 1:** Names of viruses and sera.

Serum	Year Serum Collected	HAI Titer toward Vaccine Antigen*	Victoria-Lineage Virus (passage number)	Yamagata-Lineage Virus (passage number)
none	N/A	N/A	B/CO/06/2017 (1)	B/Phuket/3073/2013 (1)
F09	2003	10	COF09 (4)	PKF09 (4)
M50	2003	40	COM50 (4)	PKM50 (3)
M74	2003	320	COM74 (3)	PKM74 (3)
M09	2011	10	COM09 (4)	PKM09 (4)
M56	2011	40	COM56 (3)	PKM56 (4)
F16	2011	320	COF16 (1)	PKF16 (3)

The name of each serum used in the passage of virus, and the name of the resulting virus with passage number (in parentheses) is shown. N/A, not applicable; HAI, hemagglutination inhibition titer; * sera were collected from subjects that had received a trivalent vaccine containing a Victoria-lineage antigen in each of the two preceding seasons. Sera collected in 2003 was from subjects vaccinated with B/Hong Kong/330/2001, and sera collected in 2011 was from subjects vaccinated with B/Brisbane/60/2008. The vaccine viruses are antigenically and temporally distinct from the passaged viruses.

Data for polymorphisms present at greater than 5% in the sequencing reads from each segment for all the viruses are presented in excel files (S1–S8 Files). The different types of mutation (transitions, transversion, synonymous and nonsynonymous) were identified and frequencies were calculated using the formulas available in excel. We did not include the short conserved noncoding regions at each end of the genomic segments that allow pairing of the 5’ and 3’ ends and are used in transcription and replication. The ratio of nonsynonymous to synonymous (dN/dS) mutations was determined for each open reading frame. The results from the major ORFs were used for comparison of ratios for genomic segment.

## Results

One IBV virus from each lineage was propagated in the presence of human sera in MDCK cells. RNA was extracted, amplified and sequenced using NGS as previously described [41]. Each parental virus was passaged in MDCK cells in parallel once without sera and the genomes sequenced using NGS. The names of the resulting viruses and passage numbers are listed in Table 1.

**Fig 1:**
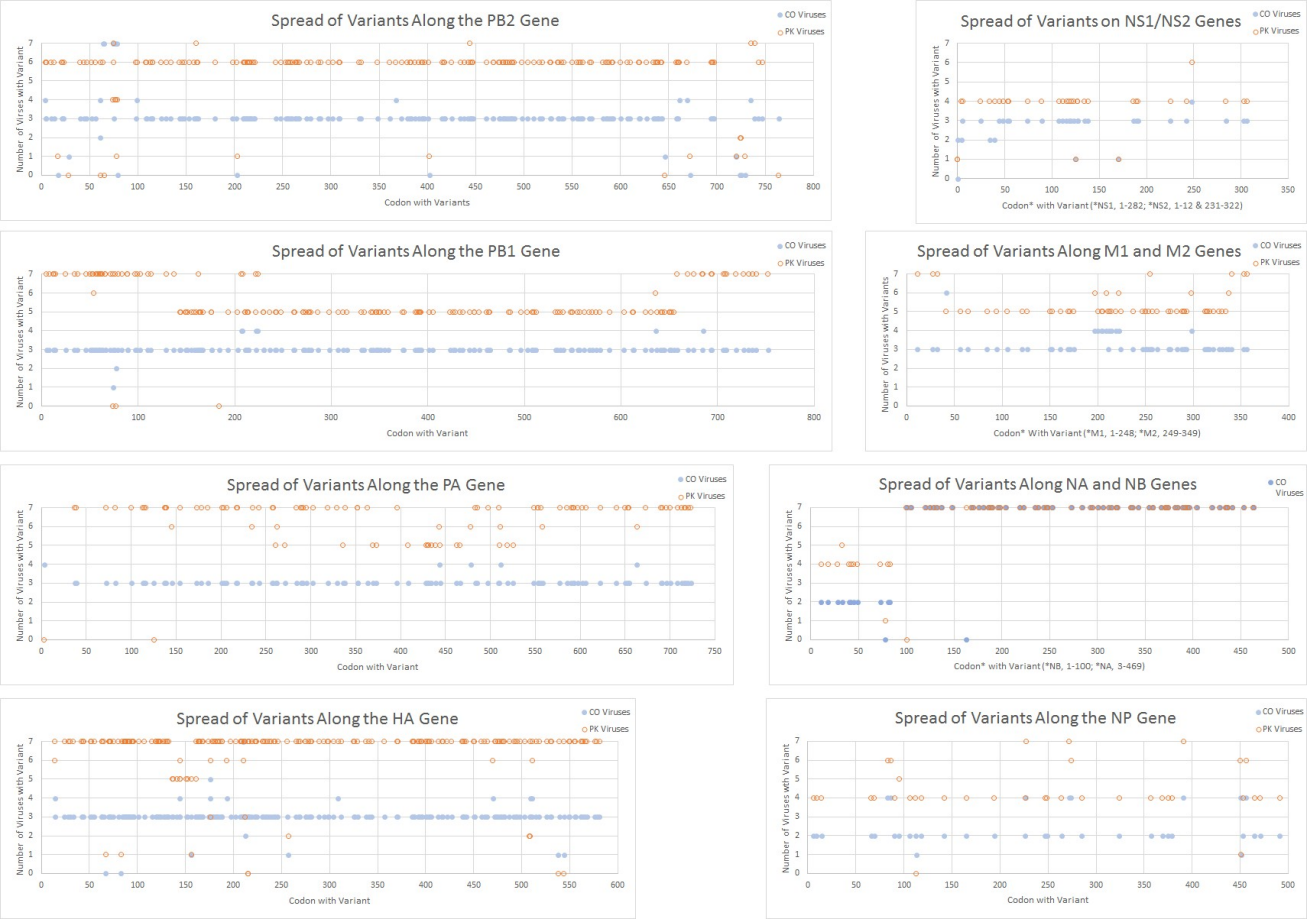


The sequences were aligned and nucleotide variants comprising more than 5% of the population were annotated (S9 File). There were differences in the composition of the genomes of the escape viruses. The number of loci with variant nucleotides differed among the viruses (S1 Fig). The spread of the variant loci across the segments is shown in Fig 1. There are several notable differences in the spread of variants among the genomic segments. First, we observed the presence in variants in more of the Yamagata-lineage escape viruses (Fig 1). Three Victoria-lineage escape viruses (COM74, COF16 and COM09) exhibited little genomic change and were similar to the parental strain. Variant nucleotides increased in abundance at more than 700 loci in the other three Victoria-lineage viruses compared to the parental virus B/CO (S1 Fig). Variant nucleotides increased in abundance at more than 200 loci in each of the Yamagata-lineage viruses. A similar number of loci decreased in abundance in each of the Yamagata-lineage escape viruses (S1 Fig). Most of the loci that changed were in the same position in the viruses from both lineages (Fig 1 and S9 File). Thus, in this experiment the Yamagata-lineage escape viruses presented as a more homogeneous group than the Victoria-lineage escape viruses but the loci that changed were similar in both groups. Secondly, we observed differences in the spread of variants along the genomic segments. This was most obvious in the genomic segments encoding proteins from more than one ORF. There were fewer variants along the NB, M1 and NS2 ORFs than the NA, M2 and NS1 ORFs (Fig 1). For the Yamagata-lineage viruses there were also portions of ORFs where variants were present at the same loci in all the viruses and sections where variants were only present in a subset of viruses. This is most apparent for the PB1 and PA genomic segments (Fig 1).

**Fig 2:**
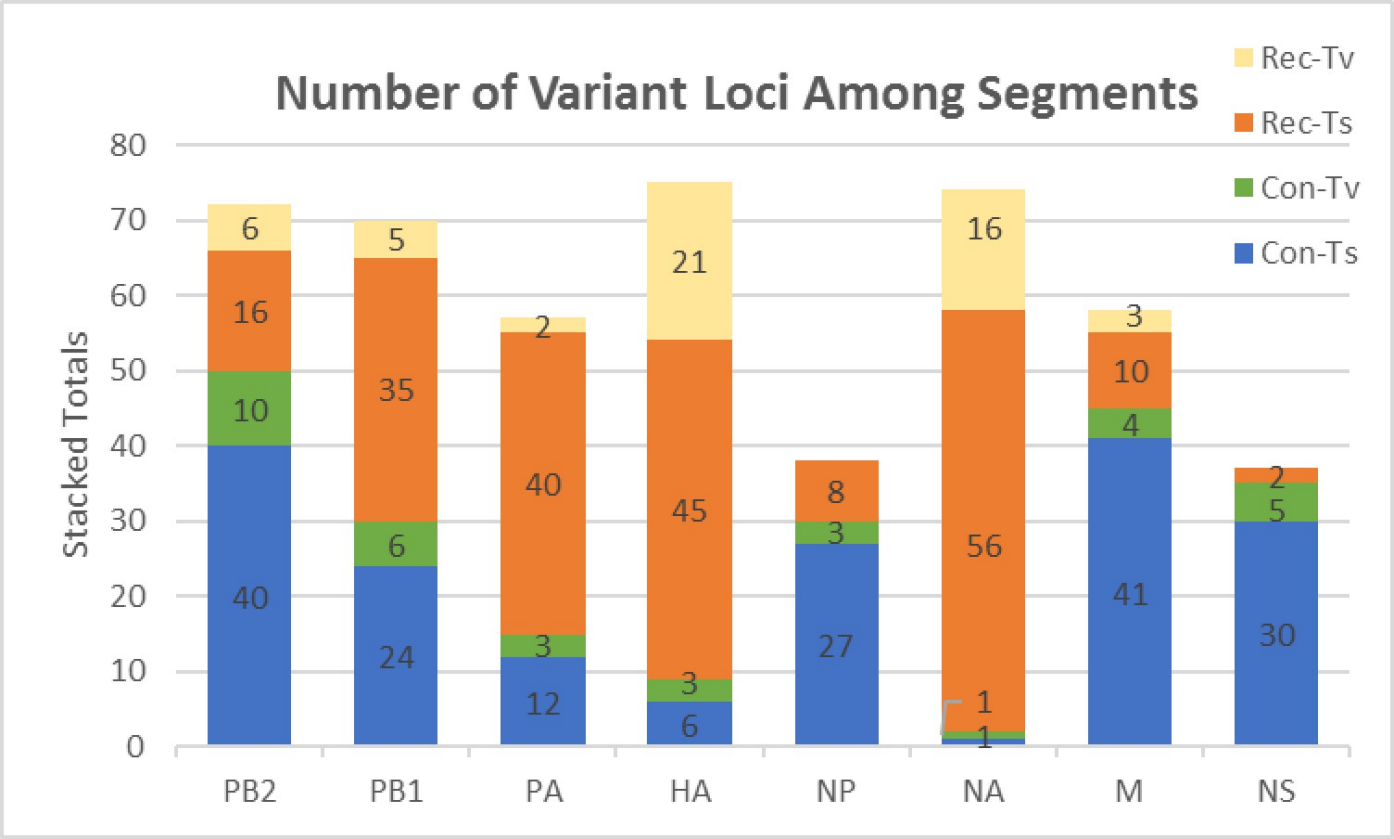
Number of variant loci among segments. The number of loci with variants present at levels greater than 5% in NGS data for ORFs in each segment are graphed. The loci are labelled as reciprocal or convergent as described in the text. Loci are further divided as either transitions (Ts) or transversions (Tv).

A qualitative analysis of the HA segment indicated that variants were present at the same loci in viruses from both lineages [41]. We analyzed the variants in the remainder of the genomic segments and observed a similar pattern. We calculated the percentage of variants that were transitions or transversions (82% and 18% respectively; Fig 2). The variants could be further classified into two broad groups, convergent and reciprocal. When the variants from each lineage gave rise to the same nucleotide we called them convergent and when the variants resulted in the loci from the virus of one lineage resembling the other lineage we called them reciprocal. For the viruses analyzed in this study 55% of the loci had reciprocal changes and 45% were convergent. The percentage of loci with reciprocal changes differed widely among the segments. Only 5% of the changes were reciprocal in the NS segment but more than 88% of changes for the HA and NA segments were reciprocal. The range was 21–57% for the remaining segments. The number and type of each variant is shown in Fig 2.

**Fig 3:**
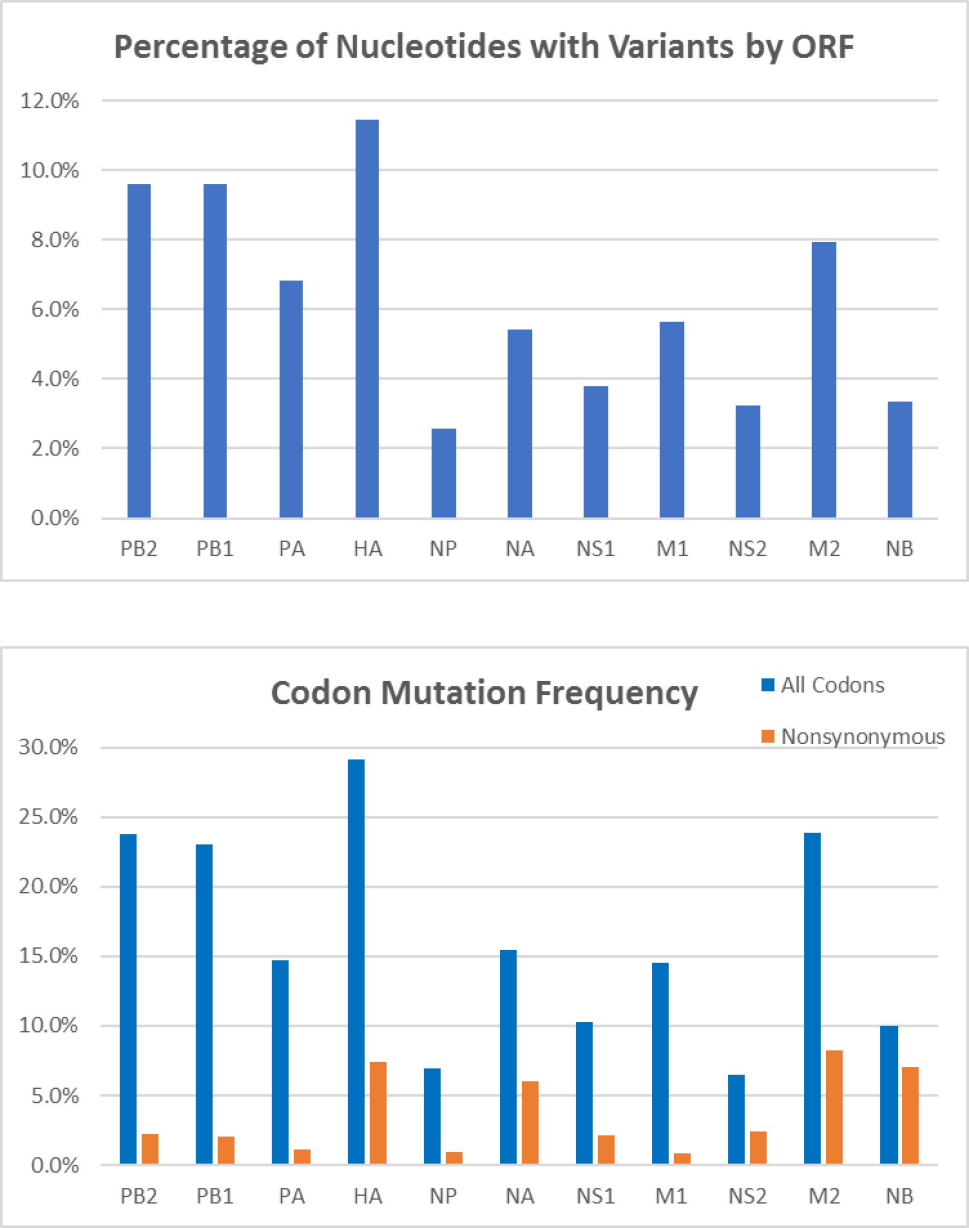
Frequency of variants among ORFs and codons. **A;** The percentage of nucleotides containing variants at levels greater than 5% is graphed for each ORF. **B;** The percentage of codons containing variants is graphed for each ORF. The percentage of codons with variants that result in nonsynonymous changes is graphed.

We analyzed the variants in each ORF. The percentage of nucleotides in each ORF with variants was determined (Fig 3A). The frequency of variants was not spread evenly across the ORFs: the HA ORF had the highest percentage of nucleotides with variants detected and the NP ORF had the lowest. We calculated the percentage of nucleotides and codons changed for each ORF throughout the genome (Fig 3). Nucleotide mutations were most frequent in the HA ORF (11.4%) and least frequent in the NP ORF (2.6%). The shorter overlapping ORFs (NS1, NS2 and NB) also had a low percentage of mutated nucleotides (3.3–3.8%). The frequency of mutated codons followed a similar pattern: 29% of codons in the HA ORF, 7% for the NP ORF, and 6.5–10.3% for NS1, NS2 and NB. Interestingly, the percentage of nucleotide and codon changes for the M1 and M2 ORFs, which are in the same segment, differed. Only the termination codon for the M1 ORF and the initiation codon of the M2 ORF overlap. The percentage of nucleotides that changed in M1 and M2 was 5.6% and 8.0% respectively and the percentage of codons mutated was 14.5% and 23.9% respectively.

We assessed the effect the variants had on the coding sequences. The percentage of codons that were changed by the variant nucleotides and resulted in nonsynonymous changes was determined (Fig 3B). The spread of mutations across the genome is not even: the frequency of nonsynonymous changes is higher in the ORFs encoding the membrane proteins (HA, NA, M2 and NB) and lower in the other ORFs. There was no trend between length of the ORF. The frequency of mutations varied from just 0.8% for the M1 ORF to 8.3% for the M2 ORF. The only overlap in these two ORFs is the pentameric sequence that includes the M1 termination codon and the M2 translation initiation codon. The frequency of nonsynonymous polymorphisms in the M2 ORF (8.3%) is higher than in any other ORF (0.8–2.4%). This suggests that, because another ion channel is encoded by NB, the M2 ORF may be more susceptible to mutation.

Nonsynonymous changes can affect protein structure and could therefore be indicative of evolutionary pressures related to the viral proteins. Sixty-three nonsynonymous changes were detected in the genomes of the B/PK and B/CO viruses in this work. All were unique loci, but some conservative changes were more frequent: 12 aliphatic valine/isoleucine changes and 10 basic lysine/arginine changes. What is apparent in our analysis was that polymorphisms appeared independently at the same loci in viruses from both lineages (S1 Fig). There is also considerable overlap between the polymorphic sites identified here and those described in natural isolates as being under selective pressure (S9 File). Chen and Holmes analyzed the 102 full length IBV genomes available in 2007 [46]. Of the 17 positively selected codon positions identified by Chen and Holmes, 12 loci were identified in this work as variable (S9 File). Whole genome analyses were performed to gain insight into the evolutionary history of IBV strains responsible for a large outbreak in Taiwan in 2011–2012 [47]. Of the 33 amino acid changes identified in multiple IBV genotypes by Yang et al., 25 were identified as variable in this work (S9 File). Langat et al., analyzed the 2651 complete IBV genomes sampled globally from 1987 to 2015 [48]. They highlighted nonsynonymous substitutions on the trunk of phylogenetic trees which indicate that changes become fixed across seasons and are less likely to be passage artifacts. Of the 40 nonsynonymous substitutions for the PB2, PB1, PA, NP, NS1 and M1 genes identified by Langat et al., 32 are present in this analysis (S9 File). The recent analysis of more than 12000 IBV genomes by Virk et al., identified 22 positively selected sites, 10 of which are variable in this work [49]. Fifty-nine of the 106 substitutions identified on the trunk of the phylogenetic trees by Virk et al., are also variable in this work (S9 File). Another recent analysis of IBV strains in Vietnam identified 8 positively selected sites in the HA and NA genes, 6 of which are variable in this work [50]. Yoshihara et al., also identified 80 negatively selected sites in the HA and NA genes, 65 of which are variable in this work (S9 File). The substantial overlap of nonsynonymous variants in our work and the mutations identified in the work of others using different approaches or datasets is interesting, as is the temporal overlap with the identification of these sites both prior to and after the isolation of B/PK and B/CO. We conclude from this that the location of the variable sites in the quasispecies generated by in vitro passaging of virus is not completely random. These data suggest that most of the IBV proteins are under strong selective pressure that limits the range of mutations that the proteins can tolerate.

Synonymous substitutions (dS) are thought to be neutral, and nonsynonymous substitutions (dN) may represent selection. The dN/dS ratio is sometimes used to detect selection but that is more suited to distantly diverged sequences [51] and is also sensitive to reselection [52]. There are nonsynonymous changes in HA proteins that appear more than once as trunk mutations on phylogenetic trees [48, 49]. The loci that change from one amino acid to another and back suggest a swing in the equilibrium between genetic drift and replenishment of a naïve host population, and a limitation in the range of acceptable amino acids at that loci. We compared the dN/dS ratio for each genomic segment with the work of Virk et al., and Chen & Holmes [46, 49]. When they compared the dN/dS ratios for IBV genomic segments, they reported higher ratios for the segments encoding HA and NA. As expected, the ratios for the HA and NA coding regions were also higher in our study (Table 2). The HA and NA proteins are the main targets of the host antibody response and the regions of the genome encoding these proteins are expected to be under more selective pressure.

**Table 2:** Comparison of dN/dS ratios.

Segment	This study	Chen and Holmes, 2008	Virk et al., 2020 (Victoria)	Virk et al., 2020 (Yamagata)
**PB2**	0.10	0.04	0.004	0.018
**PB1**	0.10	0.04	0.025	0.02
**PA**	0.08	0.07	0.034	0.035
**HA**	0.34	0.22	0.11	0.046
**NP**	0.15	0.07	0.05	0.038
**NA**	0.64	0.20	0.23	0.26
**M**	0.06	0.03	0.04	0.005
**NS**	0.26	0.29	0.34	0.21

The ratio for non-synonymous to synonymous codon changes in the major ORF in each genomic segment was calculated using the variants identified in this study. Ratios from studies using sequence data from natural isolates are shown.

The trend for higher ratios for the HA, NA and NS segments and lower ratios in the PA, NP and M segments is maintained across the datasets. The ratios for the PB2 and PB1 segments in this work are relatively higher. This may indicate that there are different selective pressures on the escape viruses grown in vitro compared to natural isolates. The previously published ratios are derived from a much larger number of sequences and our ratios may be skewed by the small number of viruses analyzed.

## Discussion

### Parallel evolution and antibodies

We observed that passage of influenza B virus in tissue culture with antibodies resulted in parallel evolution in IBVs from two different lineages. Variants arose independently at the same loci in daughter populations for both the Yamagata-lineage B/PK and Victoria-lineage B/CO viruses. Parallel evolution in the HA gene has previously been observed after passage of influenza A virus in mouse lungs [53]. More in depth analyses of influenza A viruses passaged in mice demonstrated that single nucleotide variants associated with adaptation were present in all genomic segments [22, 54]. Our prior analysis of the HA gene demonstrated parallel evolution in IBV [41]. Here we interrogate the quasispecies population for each of these viruses and document parallel evolution in all the genomic segments. While the majority of the variants described did not become fixed (>50%) in the population, parallel evolution at particular loci strongly suggests evolutionary pressures are in play. Analysis of the influenza A virus genome has identified loci that are hotspots for nucleotide substitution [55]. Here we highlight loci in IBV that are more variable than other loci.

In our system we observed more diversity in the B/PK strain quasispecies passaged without sera than in the B/CO strain. Although mutations arose at the same loci in both the B/PK and B/CO escape viruses, they were present in a greater proportion of the B/PK escape viruses. Also, we report more instances of decreases in the proportion of a variants in the B/PK escape virus populations, which is likely a reflection of abundance of variants in the parental virus preparations.

We considered the effect the antibodies in the sera may have had on the evolution of the IBVs in our study. The sera used in this work were from subjects vaccinated in either 2003 or 2011 with trivalent inactivated vaccine containing a Victoria-lineage antigen. The sera should not contain specific antibodies toward the B/PK and B/CO viruses used in this study as they were isolated subsequently in 2013 and 2017 respectively. Passage of the Victoria-lineage B/CO in two of the sera samples from 2003 and one serum sample from 2011 resulted in large increases in single nucleotide variants across the genome (S1 Fig). Some changes to the HA gene in viruses from each lineage corelated with the sera used [41]. Here we observed a greater percentage of nonsynonymous changes in the ORFs encoding the membrane associated proteins HA, NA, M2 and NB suggesting these ORFs were more tolerant of mutation. It has been shown that different regions of the influenza A genome have a higher tolerance for mutation than others [56, 57]. However, as most of the variants in the NA, M2 and NB ORFs did not become fixed in the quasispecies population it suggests that, in our experimental setup, antibody pressure was not the only factor driving change in these segments.

The changes could be the result of adaptation toward MDCK cells, or specific culture conditions such as the inclusion of sera. The MDCK cells do not have a robust Mx1 and Mx2 response to influenza and this has been correlated with changes in the polymerase and NS segments [58]. While we observed changes across these genes when viruses were propagated with sera, most would not result in nonsynonymous changes and were also present in the viral quasispecies not passaged in the presence of sera. In the polymerase genes the percentage of nucleotides and the codons with variants was higher than that for most other genes (except HA and M2) indicating more malleability of the polymerase segments (Fig 3). The percentage of variants was lower for NP indicating that the NP segment is less susceptible to mutation. Further study of convergent nonsynonymous changes may identify loci important for growth in culture, or in culture with sera.

### Protein coding and genomic constraints

There are many constraints on the sequence of influenza A genomes. Some changes result in changes to protein coding and translation, some changes affect elements that control transcription and replication, while others may affect the stability of the genome. The ends of the RNA genomic segments of influenza viruses form secondary structures for replication [59]. Interrogation of influenza A genome codon usage has also been used to provide evidence of packaging signals [60]. Structures within the genomic segments are involved in interactions between segments [61]. It is likely that similar constraints also limit the diversity of IBV genomes. An association between RNA folding and the evolution of influenza A polymerase genes has also been demonstrated [62]. We restricted our analysis to ORFs but it is likely that many of the other genomic features overlap with the ORFs.

Stochastic processes, those generating random change, do not appear to be the drivers of the genomic changes observed in our study. Our experiment used a short passage time in cell culture. This may have produced a population in which deleterious mutations were maintained by the presence of more fit members of the population [63]. The presence of antibodies may have inhibited infection of the cell culture by particular serotypes further facilitating the expansion of less genetically robust viruses. However, these explanations are insufficient to explain the range of variants observed. For example, there are many codons for which the range of synonymous codons is not sampled in the quasispecies and we did not detect variants encoding nonsense mutations.

### Mutational biases

Considering only polymerase fidelity we would expect to see the same type of changes (transitions for example) to be distributed evenly across the genome. When Lyons & Lauring analyzed the impact selection has on the transition:transversion (ratio) bias in viruses they concluded that selection is a major contributor to the ratio [19]. Pauly et al., noted that sequencing clones results in biases toward mutations with minimal fitness impacts [64]. They determined the mutation rate for the 12 types of nucleotide substitutions in influenza. The mutation rates for the genetically engineered PR8 (H1N1) and Hong Kong 2014 (H3N2) viruses were generally biased toward transitions with higher rates for A to G and U to C. The mutation rates for the other transitions (G to A and C to U) were similar to the more common transversions (G to U, U to G and U to C). In contrast to Pauly et al. [64], we observed that A to C, C to A and A to T changes were more frequent among the transversions, and that the frequency of any transversion was lower than the transition frequencies. However, we note that none of the polymorphisms in the internal genes studied in this work became dominant in the population, nor did we analyze the frequency of the polymorphisms across the viral populations.

Nonsynonymous codon changes are more frequent in the ORFs encoding the four membrane associated proteins; hemagglutinin (HA), neuraminidase (NA) and two ion channel proteins (M2 and NB). The frequency of nonsynonymous codon changes portrayed a different pattern. The frequency was higher for the membrane protein-encoding ORFs (HA, NA, M2 and NB; 6.0–8.3%) than the other ORFs (0.8–2.4%). This correlates well with the experimental set up in which virus were propagated in the presence of antibodies.

There are several ways our experimental system does not replicate natural evolution. It has been predicted that in RNA viruses, where there is a large mutational load, there may be less selection during infection [24]. However, analysis of in vivo IBV infections indicates that there are stringent bottlenecks between hosts, meaning that only a small number of viral particles were responsible for establishing the infection in a new host [65]. A similar observation was made regarding influenza A infections and many of the variants detected within hosts are not detected in viral phylogeny [66, 67]. Unlike natural infections the viral load in each passage in our experiments was controlled. This was probably more supportive of quasispecies amplification than is observed in natural infections. The viruses in our experiment did not have to deal with a sialic acid rich mucosal layer. Any bottlenecks at the infectious stage would be caused by antibodies, not mucus and the associated viral enzymatic activity. If the ratio of hemagglutinin to neuraminidase, and the respective activities, was important for infectivity it may not be accurately reflected in this system [68]. Finally, MDCK cells do not have a robust Mx1 and Mx2 response to influenza [58]. Therefore, some viruses that may have been restricted by the innate immune response in a healthy individual could have flourished in vitro.

It has been hypothesized that the pyrimidine/purine content and stability of negative RNA virus genomes in complex with the nucleoprotein modulates the polymerase activity, and this then affects which loci are subject to mutation [69]. A corollary to this is that NP would tolerate fewer mutations. In this work we observed relatively few nucleotide changes to the NP segment and a low dN/dS ratio which suggests using the IBV NP for vaccine development might be a good approach. Research in mice has shown that mucosal vaccination using recombinant adenovirus encoding IBV nucleoprotein provided protection against both IBV lineages and influenza A virus [70]. Additional vaccines using conserved IBV NP epitopes are being developed [71]. The ectodomain of the M2 protein is a conserved protein across influenza A viruses making it a good target for a universal vaccine [72]. There are universal vaccines in the clinical trial phase that use both the NP and M2 proteins from influenza A [37, 40]. In IBV there is a second ORF in the NA segment that encodes a redundant protein analogous to M2 [73]. Here we report higher dN/dS ratios for the M2 and NB ORFs compared to the other IBV ORFs which may indicate that these IBV proteins are not ideal targets for a universal vaccine.

The methodology of our study presents some advantages and disadvantages. We have focused on two viruses passaged in cell culture and do not know if the quasispecies of other IBVs are similar. We have information about the genomes of these virus after passage with and without sera and have been able to document parallel evolution. Some of this evolution is likely due to the presence of antibodies during viral passage but there is no clear relationship between each of the variants described and the growth conditions. Therefore, we cannot definitively classify them as culture-induced or antibody-induced mutations. We have cataloged the presence of synonymous and nonsynonymous mutations in the viral populations. Knowledge about which minor variants are present in the parental viral strains provides an indication of what changes are possible even if they don’t become dominant. However, this makes it difficult to link changes in variant frequency to selective pressure. Variants not present in the parental strain that emerge are likely to be under strong selective pressure, whereas variants that fluctuate in frequency are likely to be under neutral pressure. Lastly, we do not investigate epistatic relationships here. Most of the polymorphisms described in this work did not become fixed and passage of the viruses at a low MOI would be required to identify epistatic relationships or mutations that improve viral fitness.

## Conclusion

This analysis of NGS data from IBVs from different lineages grown in culture in the presence of human sera provides insight about the constraints associated with IBV genome mutation. We hypothesize that variants are limited to certain loci because features of the genome prevent other mutations from arising. The presence of reciprocal variants in viruses from two different lineages, and the fixation of some of the variants as positively selected codons in natural isolates, or as trunk mutations on phylogenetic trees in contemporary and historical isolates, support this hypothesis. Further experimental testing of this hypothesis is warranted. Our data also indicates that some selective pressures apply to the whole genome while others only to certain genomic segments or parts of segments. This is exemplified by the difference in frequency of variants and the different dN/dS ratios for M1 and M2 which reside on the same genomic segment. The complexities associated with negative strand RNA virus replication make further dissection of these selective pressures difficult. However, our analysis does suggest that data mining, taking into account the experimentally demonstrated constraints on IBV genomes, may help in the design of new vaccines or antivirals.

## Supporting information

S1 File NGS for PB2. The excel file contains the aligned variant sites for the ORF of the PB2 segment for two parental influenza B viruses and the six escape viruses generated for each. A second tab shows the spread of the variants along the ORF.
(XLSX)

S2 File NGS for PB1. The excel file contains the aligned variant sites for the ORF of the PB1 segment for two parental influenza B viruses and the six escape viruses generated for each. A second tab shows the spread of the variants along the ORF.
(XLSX)

S3 File NGS for PA. The excel file contains the aligned variant sites for the ORF of the PA segment for two parental influenza B viruses and the six escape viruses generated for each. A second tab shows the spread of the variants along the ORF.
(XLSX)

S4 File NGS for HA. The excel file contains the aligned variant sites for the ORF of the HA segment for two parental influenza B viruses and the six escape viruses generated for each. A second tab shows the spread of the variants along the ORF.
(XLSX)

S5 File NGS for NP. The excel file contains the aligned variant sites for the ORF of the NP segment for two parental influenza B viruses and the six escape viruses generated for each. A second tab shows the spread of the variants along the ORF.
(XLSX)

S6 File NGS for NA. The excel file contains the aligned variant sites for the NA and NB ORFs of the NA segment for two parental influenza B viruses and the six escape viruses generated for each. A second tab shows the spread of the variants along the ORF.
(XLSX)

S7 File NGS for M. The excel file contains the aligned variant sites for the M1 and M2 ORFs of the M segment for two parental influenza B viruses and the six escape viruses generated for each. A second tab shows the spread of the variants along the ORF.
(XLSX)

S8 File NGS for NS. The excel file contains the aligned variant sites for the NS1 and NS2 ORFs of the NS segment for two parental influenza B viruses and the six escape viruses generated for each. A second tab shows the spread of the variants along the ORF.
(XLSX)

S9 File Alignment of B/CO and B/PK open reading frames. The open reading frames for each genomic segment from the B/Colorado/06/16 (CO/17) and B/Phuket/3073/2013 (PK/13) viruses are aligned. Nucleotides that differ are shaded in gray it the coding sequence remains the same (synonymous) and in yellow if the difference results in a difference in amino acid (nonsynonymous). Additional residues of interested described in other publications are highlighted and annotated at the end of the section for each genomic segment.
(DOCX)

S1 Fig Number of Loci with variation >5% in escape viruses. The number of loci with variants present at levels greater than 5% in the NGS data are graphed for each escape virus. The viruses identified with CO are derived from the Victoria-lineage virus B/Colorado/06/2017 and the viruses with PK are derived from the Yamagata-lineage virus B/Phuket/3073/2013. Blue bars indicate loci where a variant increased in frequency compared to that in the virus grown without antibodies, orange bars indicate loci where a variant decreased in frequency compared to that in the virus grown without antibodies.
(DOCX)
