# Supplementary material for: Insights from the comparison of genomic variants from two influenza B viruses grown in the presence of human antibodies in cell culture
Source: PLoS One. 2020 Sep 14;15(9):e0239015. doi: 10.1371/journal.pone.0239015 (PMC7489522; doi:10.1371/journal.pone.0239015)
Supplement: S9 File — The open reading frames for each genomic segment from the B/Colorado/06/16 (CO/17) and B/Phuket/3073/2013 (PK/13) viruses are aligned. Nucleotides that differ are shaded in gray it the coding sequence remains the same (synonymous) and in yellow if the difference results in a difference in amino acid (nonsynonymous). Additional residues of interested described in other publications are highlighted and annotated at the end of the section for each genomic segment. (DOCX) [file pone.0239015.s009.docx]

**Supplementary File 9. Alignment of B/CO and B/PK Open Reading Frames**

The open reading frames for each genomic segment from the B/Colorado/06/16 (CO/17) and B/Phuket/3073/2013 (PK/13) viruses are aligned. Nucleotides that differ are shaded in gray it the coding sequence remains the same (synonymous) and in yellow if the difference results in a difference in amino acid (nonsynonymous). Identical nucleotides with variants present in both viruses are also shaded gray. Additional residues of interested described in other publications are highlighted and annotated at the end of the section for each genomic segment.

**PB2**

1 M T L A K I E L L K Q L L R D N E A K T V 21

CO/17 11 ATG ACA TTA GCC AAG ATT GAA TTG TTA AAA CAA CTG CTA AGG GAC AAT GAG GCC AAA ACA G 70

||| ||| || || || ||| ||| ||| ||| || ||| ||| || ||| ||| ||| || ||| ||| ||| |

PK/13 2 ATG ACA TTG GCT AAA ATT GAA TTG TTA AAG CAA CTG TTA AGG GAC AAT GAA GCC AAA ACA G 61

22 L K Q T T V D Q Y N I I R K F N T S R I 41

CO/17 71 TT TTG AAG CAA ACA ACA GTA GAC CAA TAT AAC ATA ATA AGA AAA TTC AAT ACA TCA AGG A 130

| || || ||| ||| ||| ||| || ||| ||| ||| ||| ||| ||| ||| ||| ||| ||| ||| || |

PK/13 62 TA CTG AAA CAA ACA ACA GTA GAT CAA TAT AAC ATA ATA AGA AAA TTC AAT ACA TCA AGA A 121

42 E K N P S L R M K W A M C S N F P L A L 61

CO/17 131 TT GAA AAG AAT CCT TCA CTA AGG ATG AAG TGG GCC ATG TGT TCT AAC TTT CCC TTG GCT C 190

|| ||| ||| || ||| ||| || ||| ||| ||| ||| || ||| ||| ||| || ||| ||| ||| |||

PK/13 122 TT GAA AAG AAC CCT TCA TTA AGG ATG AAG TGG GCG ATG TGT TCT AAT TTT CCC TTG GCT T 181

62 T K G X X A N R I P L E Y K G I Q L K T 81

CO/17 191 TA ACC AAG GGC GAA AAG GCA AAC AGA ATC CCC TTG GAA TAC AAA GGA AAA CAA CTT AAA A 250

| ||| ||| || || | | ||| ||| ||| ||| ||| ||| ||| ||| ||| ||| | | ||| ||| ||| |

PK/13 182 TG ACC AAG GGT GAC ATG GCA AAC AGA ATC CCC TTG GAA TAC AAA GGA ATA CAA CTT AAA A 241

82 N A E D I G T K G Q M C S I A A V T W W 101

CO/17 251 CA AAT GCT GAA GAC ATA GGA ACC AAA GGC CAA ATG TGC TCA ATA GCA GCA GTT ACT TGG T 310

|| ||| ||| ||| ||| ||| ||| ||| ||| ||| ||| ||| ||| ||| ||| ||| ||| ||| ||| ||| |

PK/13 242 CA AAT GCT GAA GAC ATA GGA ACC AAA GGC CAA ATG TGC TCA ATA GCA GCA GTT ACT TGG T 301

102 N **T** Y G P I G **D** T E G F E R V Y E S F F 121

CO/17 311 GG AAT ACA TAT GGA CCA ATA GGG GAC ACT GAA GGT TTT GAA AGG GTC TAC GAA AGC TTT T 370

|| ||| ||| ||| ||| ||| ||| || ||| ||| ||| ||| ||| ||| ||| ||| ||| ||| ||| ||| |

PK/13 302 GG AAT ACA TAT GGA CCA ATA GGA GAC ACT GAA GGT TTT GAA AGG GTC TAC GAA AGC TTT T 361

122 L R K M R L D N A T W G R I T F G P V E 141

CO/17 371 TT CTC AGA AAA ATG AGA CTT GAC AAC GCC ACT TGG GGC CGG ATA ACT TTT GGC CCA GTT G 430

|| ||| ||| ||| ||| ||| ||| ||| ||| ||| ||| ||| ||| ||| ||| ||| ||| ||| ||| ||| |

PK/13 362 TT CTC AGA AAA ATG AGA CTT GAC AAC GCC ACT TGG GGC CGG ATA ACT TTT GGC CCA GTT G 421

142 R V R K R V L L N P L T K E M P P D E A 161

CO/17 431 AA AGA GTG AGA AAA AGA GTA CTA CTA AAC CCT CTC ACC AAG GAA ATG CCT CCG GAT GAG G 490

|| ||| ||| ||| ||| ||| ||| ||| ||| ||| ||| ||| ||| ||| ||| ||| ||| ||| ||| ||| |

PK/13 422 AA AGA GTG AGA AAA AGA GTA CTA CTA AAC CCT CTC ACC AAG GAA ATG CCT CCG GAT GAG G 481

162 S N V I M E I L F P K E A G I P R E S T 181

CO/17 491 CA AGC AAT GTG ATA ATG GAA ATA TTG TTC CCT AAA GAA GCA GGA ATA CCA AGA GAA TCC A 550

| ||| ||| ||| ||| ||| ||| ||| ||| ||| ||| ||| ||| ||| ||| ||| ||| ||| ||| ||| |

PK/13 481 CG AGC AAT GTG ATA ATG GAA ATA TTG TTC CCT AAA GAA GCA GGA ATA CCA AGA GAA TCC A 540

182 W I H R E L I K E K R E K L K G T M I T 201

CO/17 551 CT TGG ATA CAT AGG GAA CTG Ata aaa gaa aaa aga gaa aaa TTG AAA GGA ACA ATG ATA A 610

|| ||| ||| ||| ||| ||| ||| ||| ||| ||| ||| ||| ||| ||| ||| ||| ||| ||| ||| ||| |

PK/13 541 CT TGG ATA CAT AGG GAA CTG ATA AAA GAA AAA AGA GAA AAA TTG AAA GGA ACA ATG ATA A 600

202 P I V L A Y M L E R E L V A R R R F L P 221

CO/17 611 CT CCA ATC GTA CTG GCA TAC ATG CTT GAA AGA GAA CTG GTT GCT CGA AGA AGA TTC TTG C 670

|| ||| ||| ||| ||| ||| ||| ||| ||| ||| ||| ||| ||| ||| ||| ||| ||| ||| ||| ||| |

PK/13 601 CT CCA ATC GTA CTG GCA TAC ATG CTT GAA AGA GAA CTG GTT GCT CGA AGA AGA TTC TTG C 660

222 V A G A T S A E F I E M L H C L Q G E N 241

CO/17 671 CA GTG GCA GGA GCA ACA TCA GCT GAG TTC ATA GAA ATG CTA CAC TGC TTA CAA GGT GAA A 730

|| ||| ||| ||| ||| ||| ||| ||| ||| ||| ||| ||| ||| ||| ||| ||| ||| ||| ||| ||| |

PK/13 661 CA GTG GCA GGA GCA ACA TCA GCT GAG TTC ATA GAA ATG CTA CAC TGC TTA CAA GGT GAA A 720

242 W R Q I Y H P G G N K L T E S R S Q S M 261

CO/17 731 AT TGG AGA CAA ATA TAT CAC CCA GGA GGG AAT AAA TTA ACT GAG TCC AGG TCT CAA TCA A 790

|| ||| ||| ||| ||| ||| ||| ||| ||| ||| ||| ||| ||| ||| ||| ||| ||| ||| ||| ||| |

PK/13 721 AT TGG AGA CAA ATA TAT CAC CCA GGA GGG AAT AAA TTA ACT GAG TCC AGG TCT CAA TCA A 780

262 I V A C R K I I R R S I V A S N P L E L 281

CO/17 791 TG ATA GTA GCT TGT AGG AAA ATA ATC AGA AGA TCA ATA GTC GCT TCA AAC CCA CTG GAG C 850

|| ||| ||| ||| ||| ||| ||| ||| ||| ||| ||| ||| ||| ||| ||| ||| ||| ||| ||| ||| |

PK/13 781 TG ATA GTA GCT TGT AGG AAA ATA ATC AGA AGA TCA ATA GTC GCT TCA AAC CCA CTG GAG C 840

282 A V E I A N K T V I D T E P L K S C L A 301

CO/17 851 TA GCT GTA GAA ATT GCA AAC AAG ACT GTG ATA GAT ACT GAA CCT TTA AAG TCA TGT CTG G 910

|| ||| ||| ||| ||| ||| ||| ||| ||| ||| ||| ||| ||| ||| ||| ||| ||| ||| ||| ||| |

PK/13 841 TA GCT GTA GAA ATT GCA AAC AAG ACT GTG ATA GAT ACT GAA CCT TTA AAG TCA TGT CTG G 900

302 A I D G G D V A C D I I R A A L G L K I 321

CO/17 911 CA GCC ATA GAC GGA GGT GAT GTA GCT TGT GAC ATA ATA AGA GCT GCA TTA GGA CTA AAG A 970

|| ||| ||| ||| ||| ||| ||| ||| ||| ||| ||| ||| ||| ||| ||| ||| ||| ||| ||| ||| |

PK/13 901 CA GCC ATA GAC GGA GGT GAT GTA GCT TGT GAC ATA ATA AGA GCT GCA TTA GGA CTA AAG A 960

322 R Q R Q R F G R L E L K R I S G R G F K 341

CO/17 971 TC AGA CAA AGA CAA AGA TTT GGA CGG CTT GAG CTA AAA AGA ATA TCA GGA AGA GGA TTC A 1030

|| ||| ||| ||| ||| ||| ||| ||| ||| ||| ||| ||| ||| ||| ||| ||| ||| ||| ||| ||| |

PK/13 961 TC AGA CAA AGA CAA AGA TTT GGA CGG CTT GAG CTA AAA AGA ATA TCA GGA AGA GGA TTC A 1020

342 N D E E I L I G N G T I **Q** K I G I W D G 361

CO/17 1031 AA AAT GAT GAA GAA ATA TTA ATA GGG AAC GGA ACA ATA CAG AAG ATT GGA ATA TGG GAC G 1090

|| ||| ||| ||| ||| ||| ||| ||| ||| ||| ||| ||| ||| ||| ||| ||| ||| ||| ||| ||| |

PK/13 1021 AA AAT GAT GAA GAA ATA TTA ATA GGG AAC GGA ACA ATA CAG AAG ATT GGA ATA TGG GAC G 1080

362 E E E F H V **R** C G E C R G I L K K S K M 381

CO/17 1091 GG GAA GAG GAG TTC CAT GTA AGA TGT GGT GAA TGC AGA GGA ATA TTa aaa aag agt aaa a 1150

|| ||| ||| ||| ||| ||| ||| ||| ||| ||| ||| ||| || ||| ||| ||| ||| ||| ||| ||| |

PK/13 1081 GG GAA GAG GAG TTC CAT GTA AGA TGT GGT GAA TGC AGG GGA ATA TTA AAA AAG AGT AAA A 1140

382 K L E K L L I N S **A** K K E D M R D L I I 401

CO/17 1151 tg aaa ctg gaa aaa cta ctg ata aat tca gcc aaa aaG GAG GAT ATG AGA GAT TTA ATA A 1210

|| ||| ||| ||| ||| ||| ||| ||| ||| ||| ||| ||| ||| ||| ||| ||| ||| ||| ||| ||| |

PK/13 1141 TG AAA CTG GAA AAA CTA CTG ATA AAT TCA GCC AAA AAG GAG GAT ATG AGA GAT TTA ATA A 1200

402 L C M V F S Q D T R M F Q G V R G E I N 421

CO/17 1211 TC TTA TGC ATG GTA TTT TCT CAA GAC ACT AGG ATG TTC CAA GGG GTG AGA GGA GAA ATA A 1270

|| ||| ||| ||| ||| ||| ||| ||| ||| ||| ||| ||| ||| ||| ||| ||| ||| ||| ||| ||| |

PK/13 1201 TC TTA TGC ATG GTA TTT TCT CAA GAC ACT AGG ATG TTC CAA GGG GTG AGA GGA GAA ATA A 1260

422 F L N R A G Q L L S P M Y Q L Q R Y F L 441

CO/17 1271 AT TTT CTT AAT CGA GCA GGC CAA CTT TTA TCT CCA ATG TAC CAA CTC CAA CGA TAT TTT T 1330

|| ||| ||| ||| ||| ||| ||| ||| ||| ||| ||| ||| ||| ||| ||| ||| ||| ||| ||| ||| |

PK/13 1261 AT TTT CTT AAT CGA GCA GGC CAA CTT TTA TCT CCA ATG TAC CAA CTC CAA CGA TAT TTT T 1320

442 N R S N D L F D Q W G Y E E S P K A S E 461

CO/17 1331 TG AAT AGA AGC AAC GAC CTT TTT GAT CAA TGG GGG TAT GAG GAA TCA CCC AAA GCA AGT G 1390

|| ||| ||| ||| || ||| ||| ||| ||| ||| ||| ||| ||| ||| ||| ||| ||| ||| ||| ||| |

PK/13 1321 TG AAT AGA AGC AAT GAC CTT TTT GAT CAA TGG GGG TAT GAG GAA TCA CCC AAA GCA AGT G 1380

462 L H G I N E S M N A S D Y T L K G I***** V V 481

CO/17 1391 AA CTA CAT GGG ATA AAT GAA TCA ATG AAT GCA TCT GAC TAT ACA TTG AAA GGG ATT GTA G 1450

|| ||| ||| ||| ||| ||| ||| ||| ||| ||| ||| ||| ||| ||| ||| ||| ||| ||| ||| ||| |

PK/13 1381 AA CTA CAT GGG ATA AAT GAA TCA ATG AAT GCA TCT GAC TAT ACA TTG AAA GGG ATT GTA G 1440

482 T R N V I D D F S S T E T E K V S I T K 501

CO/17 1451 TG ACA AGA AAT GTA ATT GAC GAC TTT AGC TCT ACT GAA ACA GAA AAA GTA TCC ATA ACA A 1510

|| ||| ||| ||| ||| ||| ||| ||| ||| ||| ||| ||| ||| ||| ||| ||| ||| ||| ||| ||| |

PK/13 1441 TG ACA AGA AAT GTA ATT GAC GAC TTT AGC TCT ACT GAA ACA GAA AAA GTA TCC ATA ACA A 1500

502 N L S L I K R T G E V I M G A N D V S E 521

CO/17 1511 AA AAT CTT AGC TTA ATA AAA AGG ACT GGA GAA GTC ATA ATG GGA GCT AAT GAC GTG AGT G 1570

|| ||| ||| ||| ||| ||| ||| ||| ||| ||| ||| ||| ||| ||| ||| ||| ||| ||| ||| ||| |

PK/13 1501 AA AAT CTT AGC TTA ATA AAA AGG ACT GGA GAA GTC ATA ATG GGA GCT AAT GAC GTG AGT G 1560

522 L E S Q A Q L M I T Y D T P K M W E M G 541

CO/17 1571 AA TTA GAA TCA CAA GCA CAG CTG ATG ATA ACA TAT GAT ACA CCT AAA ATG TGG GAA ATG G 1630

|| ||| ||| ||| ||| ||| ||| ||| ||| ||| ||| ||| ||| ||| ||| ||| ||| ||| ||| ||| |

PK/13 1561 AA TTA GAA TCA CAA GCA CAG CTG ATG ATA ACA TAT GAT ACA CCT AAA ATG TGG GAA ATG G 1620

542 T T K E L V Q N T Y Q W V **LL** K N L V T L 561

CO/17 1631 GA ACA ACC AAA GAA CTG GTG CAA AAC ACT TAT CAA TGG GTG CTA AAA AAC TTG GTG ACA C 1690

|| ||| ||| ||| ||| ||| ||| ||| ||| ||| ||| ||| ||| ||| ||| ||| ||| ||| ||| ||| |

PK/13 1621 GA ACA ACC AAA GAA CTG GTG CAA AAC ACT TAT CAA TGG GTG CTA AAA AAC TTG GTG ACA C 1680

562 K A Q F L L G K E D M F Q W D A F E A F 581

CO/17 1691 TG AAG GCT CAG TTT CTT CTA GGA AAA GAG GAC ATG TTC CAA TGG GAT GCA TTT GAA GCA T 1750

|| ||| ||| ||| ||| ||| ||| ||| ||| ||| ||| ||| ||| ||| ||| ||| ||| ||| ||| ||| |

PK/13 1681 TG AAG GCT CAG TTT CTT CTA GGA AAA GAG GAC ATG TTC CAA TGG GAT GCA TTT GAA GCA T 1740

582 E S I I P Q K M A G Q Y S G F A R A V L 601

CO/17 1751 TT GAG AGC ATA ATT CCT CAG AAA ATG GCT GGT CAG TAC AGT GGA TTT GCA AGA GCA GTG C 1810

|| ||| ||| ||| ||| ||| ||| ||| ||| ||| ||| ||| ||| ||| ||| ||| ||| ||| ||| ||| |

PK/13 1741 TT GAG AGC ATA ATT CCT CAG AAA ATG GCT GGT CAG TAC AGT GGA TTT GCA AGA GCA GTG C 1800

602 K Q M R D Q E V M K T D Q F I K L L P F 621

CO/17 1811 TC AAA CAA ATG AGA GAC CAG GAG GTT ATG AAA ACT GAC CAG TTC ATA AAG TTG CTG CCT T 1870

|| ||| ||| ||| ||| ||| ||| ||| ||| ||| ||| ||| ||| ||| ||| ||| ||| ||| ||| ||| |

PK/13 1801 TC AAA CAA ATG AGA GAC CAG GAG GTT ATG AAA ACT GAC CAG TTC ATA AAG TTG CTG CCT T 1860

622 C F S P P K L R S***** N G E P Y Q F L K L V 641

CO/17 1871 TT TGT TTC TCA CCA CCA AAA TTA AGG AGC AAT GGG GAG CCT TAT CAA TTC TTA AAA CTT G 1930

|| ||| ||| ||| ||| ||| ||| ||| ||| ||| ||| ||| ||| ||| ||| ||| ||| ||| ||| ||| |

PK/13 1861 TT TGT TTC TCA CCA CCA AAA TTA AGG AGC AAT GGG GAG CCT TAT CAA TTC TTA AAA CTT G 1920

642 L K G G G E N F I E V R K G S P L F S Y 661

CO/17 1931 TA TTG AAA GGA GGA GGG GAA AAT TTC ATC GAA GTA AGG AAA GGG TCT CCT CTA TTT TCC T 1990

|| ||| ||| ||| ||| ||| ||| ||| ||| ||| ||| ||| ||| ||| ||| ||| ||| ||| ||| ||| |

PK/13 1921 TA TTG AAA GGA GGA GGG GAA AAT TTC ATC GAA GTA AGG AAA GGG TCT CCT CTA TTT TCC T 1980

662 N P Q T E V L T I C G R M M S L K G K I 681

CO/17 1991 AT AAT CCA CAA ACA GAA GTC TTA ACT ATA TGT GGC AGA ATG ATG TCA TTA AAA GGG AAA A 2050

|| ||| ||| ||| ||| ||| ||| || ||| ||| || ||| ||| ||| ||| ||| ||| ||| ||| ||| |

PK/13 1981 AT AAT CCA CAA ACA GAA GTC CTA ACT ATA TGC GGC AGA ATG ATG TCA TTA AAA GGG AAA A 2040

682 E D E E R N R S M G N A V L A G F L V S 701

CO/17 2051 TT GAA GAT GAA GAA AGG AAT AGA TCA ATG GGG AAT GCA GTA TTA GCA GGC TTT CTC GTT A 2110

|| ||| ||| ||| ||| ||| ||| ||| ||| ||| ||| ||| ||| ||| ||| ||| ||| ||| ||| ||| |

PK/13 2041 TT GAA GAT GAA GAA AGG AAT AGA TCA ATG GGG AAT GCA GTA TTA GCA GGC TTT CTC GTT A 2100

702 G K Y D P D L G D F K T I E E L E K L K 721

CO/17 2111 GT GGC AAG TAT GAC CCA GAT CTT GGA GAT TTC AAA ACT ATT GAA GAA CTT GAA AAG CTG A 2170

|| ||| ||| ||| ||| ||| ||| ||| ||| ||| ||| ||| ||| ||| ||| ||| ||| ||| ||| ||| |

PK/13 2101 GT GGC AAG TAT GAC CCA GAT CTT GGA GAT TTC AAA ACT ATT GAA GAA CTT GAA AAG CTG A 2160

722 P G E K A N I L L Y Q G K P V K V V K R 741

CO/17 2171 AA CCA GGG GAA AAG GCA AAC ATC TTG CTT TAT CAA GGA AAA CCA GTT AAA GTA GTA AAA A 2230

|| ||| ||| ||| || ||| ||| ||| || ||| ||| ||| ||| ||| || ||| ||| ||| || ||| |

PK/13 2161 AA CCA GGG GAA AAA GCA AAC ATC TTA CTT TAT CAA GGA AAA CCC GTT AAA GTA GTT AAA A 2220

742 K R Y S A L S N D I S Q G I K R Q R M T 761

CO/17 2231 GG AAA AGG TAT AGT GCT TTG TCC AAT GAC ATT TCA CAA GGA ATT AAG AGA CAA AGA ATG A 2290

|| ||| || ||| ||| ||| || ||| ||| ||| ||| ||| ||| ||| ||| ||| ||| ||| ||| ||| |

PK/13 2221 GG AAA AGA TAT AGT GCT TTA TCC AAT GAC ATT TCA CAA GGA ATT AAG AGA CAA AGA ATG A 2280

762 V E S M G W A L S 770

CO/17 2291 CA GTT GAG TCT ATG GGG TGG GCC TTG AGC TAA 2311

|| ||| ||| || ||| ||| ||| ||| ||| ||| |||

PK/13 2281 CA GTT GAG TCC ATG GGG TGG GCC TTG AGC TAA 2312

Protein numbering is shown on the right side for the full length PB2 ORF.

Residues highlighted in **bold** were identified in Chen and Holmes (2008) Table 2 as positively selected amino acid positions, 1/1 PB2 were subject to mutation in this analysis.

Residues highlighted in **bold** were identified in Yang et al., (2012) Table 2 as amino acid substitutions in multiple genotypes, 1/1 PB2 were subject to mutation in this analysis.

Residues highlighted in **bold** were identified in Virk et al., (2020) supplementary information as truck amino acid substitutions on phylogenetic trees, 2/5 PB2 were subject to mutation in this analysis. Amino acid positions identified as positively selected sites are marked with an asterisk *****

**PB1**

M N I N P Y F L F I D V P V Q A A I S T T 21

CO/17 3 ATG AAT ATA AAT CCT TAT TTT CTC TTC ATA GAT GTG CCC GTA CAG GCA GCA ATT TCA ACA A 62

||| ||| ||| ||| || ||| ||| || ||| ||| ||| || ||| || ||| ||| ||| ||| ||| ||| |

PK/13 2 ATG AAT ATA AAT CCG TAT TTT CTA TTC ATA GAT GTA CCC ATA CAG GCA GCA ATT TCA ACA A 61

15 24 36 39

F P Y T G V P P Y S H G T G T G **YY** T I D 41

CO/17 63 CA TTC CCA TAC ACT GGT GTT CCC CCT TAT TCC CAT GGA ACA GGA ACA GGT TAC ACA ATA G 122

|| ||| ||| ||| || ||| ||| ||| ||| ||| ||| ||| ||| || ||| ||| || ||| ||| ||| |

PK/13 62 CA TTC CCA TAC ACC GGT GTT CCC CCT TAT TCC CAT GGA ACG GGA ACA GGC TAC ACA ATA G 121

75 111

T V I R T H E Y S N K G K Q Y I S D V T 61

CO/17 123 AC ACC GTG ATC AGA ACG CAT GAG TAC TCA AAC AAG GGG AAG CAG TAC ATT TCT GAT GTT A 182

|| ||| ||| ||| ||| || ||| ||| ||| || ||| || || || ||| || || ||| || | |

PK/13 122 AC ACC GTG ATC AGA ACA CAT GAG TAC TCG AAC AAA GGA AAA CAG TAT GTT TCT GAC ATC A 181

126 138 150 168 177

G C T M **VV** D P T N G P L P E D N E P S A 81

CO/17 183 CA GGA TGC ACA ATG GTG GAT CCA ACA AAT GGA CCA TTA CCC GAA GAT AAT GAG CCG AGT G 242

|| || || ||| ||| | ||| ||| ||| ||| || ||| ||| || ||| || ||| ||| || ||| |

PK/13 182 CA GGG TGT ACA ATG ATA GAT CCA ACA AAT GGG CCA TTA CCT GAA GAC AAT GAG CCA AGT G 241

186 196 237

Y A Q L D C V L E A L D R M D E E H P G 101

CO/17 243 CC TAT GCG CAA TTA GAT TGC GTT TTA GAG GCT TTG GAT AGA ATG GAT GAA GAA CAC CCA G 302

|| ||| || ||| ||| ||| ||| ||| | ||| ||| ||| ||| ||| ||| ||| || ||| || ||| |

PK/13 242 CC TAT GCA CAA TTA GAT TGC GTT CTG GAG GCT TTG GAT AGA ATG GAT GAG GAA CAT CCA G 301

249 265 297

L F Q A A S Q N A M E A L M V T T V D K 121

CO/17 303 GT CTT TTT CAA GCA GCC TCA CAG AAT GCT ATG GAG GCC CTA ATG GTC ACA ACT GTA GAC A 362

|| || ||| ||| ||| ||| ||| ||| ||| || ||| ||| || ||| ||| ||| ||| ||| ||| ||| |

PK/13 302 GT CTG TTT CAA GCA GCC TCA CAG AAT GCC ATG GAG GCA CTA ATG GTC ACA ACT GTA GAC A 361

304 357

L T Q G R Q T F D W T V C R N Q P A A T 141

CO/17 363 AA TTA ACC CAG GGG AGA CAG ACT TTT GAT TGG ACA GTA TGC AGA AAC CAA CCT GCT GCA A 422

|| ||| ||| ||| ||| ||| ||| ||| || ||| ||| ||| ||| ||| ||| ||| || ||| ||| ||| |

PK/13 362 AA TTA ACC CAG GGG AGA CAG ACT TTC GAT TGG ACA GTA TGC AGA AAC CAG CCT GCT GCA A 421

388 411

A L N T T I T S F R L N D L N G A D K G 161

CO/17 423 CG GCA CTG AAT ACA ACA ATA ACC TCT TTT AGG TTG AAT GAT TTA AAT GGA GCC GAC AAA G 482

|| ||| ||| ||| ||| ||| ||| ||| ||| ||| ||| ||| ||| ||| ||| ||| ||| ||| ||| ||| |

PK/13 422 CG GCA CTG AAT ACA ACA ATA ACC TCT TTT AGG TTG AAT GAT TTA AAT GGA GCC GAC AAA G 481

429 480

G L I P F C Q D I I D S L D R P E M T F 181

CO/17 483 GT GGA TTA ATA CCT TTT TGC CAG GAT ATC ATT GAT TCA TTA GAC AGA CCT GAA ATG ACT T 542

|| || ||| ||| ||| ||| ||| ||| ||| ||| ||| ||| ||| ||| ||| ||| ||| ||| ||| ||| |

PK/13 482 GT GGG TTA ATA CCT TTT TGC CAG GAT ATC ATT GAT TCA TTA GAC AGA CCT GAA ATG ACT T 541

486 528

F S V K N I K K K L P A K N R K G F **L** I 201

CO/17 543 TC TTC TCA GTA AAG AAT ATA AAG AAA AAA TTG CCT GCC AAA AAC AGA AAG GGT TTC CTC A 602

|| ||| ||| ||| ||| ||| ||| ||| ||| ||| ||| ||| ||| ||| ||| ||| ||| ||| ||| ||| |

PK/13 542 TC TTC TCA GTA AAG AAT ATA AAG AAA AAA TTG CCT GCC AAA AAC AGA AAG GGT TTC CTC A 601

552 579

K R I P M K **I** K D K I T K V E Y I K R A 221

CO/17 603 TA AAG AGG ATA CCA ATG AAA ATA AAA GAC AAA ATA ACC AAA GTG GAA TAC ATC AAA AGA G 662

|| ||| ||| ||| ||| ||| || || ||| ||| ||| ||| ||| ||| ||| ||| ||| ||| ||| ||| |

PK/13 602 TA AAG AGG ATA CCA ATG AAG GTA AAA GAC AAA ATA ACC AAA GTG GAA TAC ATC AAA AGA G 661

609 622 633 637 641(aaa-aga)

L S L N T M T K D A E R G K L K R R A I 241

CO/17 663 CA CTA TCA CTA AAC ACA ATG ACA AAA GAC GCT GAA AGA GGC AAA CTG AAA AGA AGA GCG A 722

|| || ||| || ||| ||| ||| ||| ||| ||| ||| ||| ||| ||| ||| ||| ||| ||| ||| ||| |

PK/13 662 CA TTA TCA TTA AAC ACA ATG ACA AAA GAC GCT GAA AGA GGC AAA CTG AAA AGA AGA GCG A 721

664 708

A T A G I Q I R G F V L V V E N L A K N 261

CO/17 723 TT GCC ACT GCT GGA ATA CAA ATA AGA GGG TTT GTA TTA GTA GTT GAA AAC TTG GCT AAA A 782

|| ||| ||| ||| ||| ||| ||| ||| ||| ||| ||| ||| ||| ||| ||| ||| ||| ||| ||| ||| |

PK/13 722 TT GCC ACT GCT GGA ATA CAA ATA AGA GGG TTT GTA TTA GTA GTT GAA AAC TTG GCT AAA A 781

726 744

I C E N L E Q S G L P V G G N E K K A K 281

CO/17 783 AT ATA TGT GAA AAT CTA GAA CAA AGT GGT TTA CCA GTA GGT GGA AAC GAG AAG AAA GCC A 842

|| ||| ||| ||| ||| ||| ||| ||| ||| ||| ||| ||| ||| ||| ||| ||| ||| ||| ||| ||| |

PK/13 782 AT ATA TGT GAA AAT CTA GAA CAA AGT GGT TTA CCA GTA GGT GGA AAC GAG AAG AAA GCC A 841

783 837

L S N A V A K M L S N C P P G G I S M T 301

CO/17 843 AA CTG TCA AAT GCA GTG GCC AAA ATG CTC AGT AAC TGC CCA CCA GGA GGG ATT AGC ATG A 902

|| ||| ||| ||| ||| ||| ||| ||| ||| ||| ||| ||| ||| ||| ||| ||| ||| ||| ||| ||| |

PK/13 842 AA CTG TCA AAT GCA GTG GCC AAA ATG CTC AGT AAC TGC CCA CCA GGA GGG ATT AGC ATG A 901

858 894

V T G D N T K W N E C L N P R I F L A M 321

CO/17 903 CA GTA ACA GGA GAC AAT ACA AAA TGG AAT GAA TGT TTA AAC CCA AGA ATC TTT TTG GCC A 962

|| ||| ||| ||| ||| ||| ||| ||| ||| ||| ||| ||| ||| ||| ||| ||| ||| ||| ||| ||| |

PK/13 902 CA GTA ACA GGA GAC AAT ACA AAA TGG AAT GAA TGT TTA AAC CCA AGA ATC TTT TTG GCC A 961

921 946(aga-cga) 960

T E R I T R D S P V W F R D F C S I A P 341

CO/17 963 TG ACT GAA AGA ATA ACC AGA GAC AGC CCA GTT TGG TTC AGG GAT TTT TGT AGT ATA GCA C 1022

|| ||| ||| ||| ||| ||| ||| ||| ||| ||| ||| ||| ||| ||| ||| ||| ||| ||| ||| ||| |

PK/13 962 TG ACT GAA AGA ATA ACC AGA GAC AGC CCA GTT TGG TTC AGG GAT TTT TGT AGT ATA GCA C 1021

992(gtt-att)1000(agg-cgg)

V L F S N K I A R L G K G F M **II** T S K T 361

CO/17 1023 CG GTC CTG TTC TCC AAT AAG ATA GCA AGA TTG GGG AAA GGA TTT ATG ATA ACA AGC AAA A 1082

|| ||| ||| ||| ||| ||| ||| ||| ||| ||| ||| ||| ||| ||| ||| ||| ||| ||| ||| ||| |

PK/13 1022 CG GTC CTG TTC TCC AAT AAG ATA GCA AGA TTG GGG AAA GGA TTT ATG ATA ACA AGC AAA A 1081

1028(ctg-tgt) 1077

K R L K A Q I P C P D L F S I P L E R Y 381

CO/17 1083 CA AAA AGA CTA AAG GCT CAA ATA CCT TGT CCT GAT CTG TTT AGT ATA CCA TTA GAA AGA T 1142

|| ||| ||| ||| ||| ||| ||| ||| ||| ||| ||| ||| ||| ||| ||| ||| ||| ||| ||| ||| |

PK/13 1082 CA AAA AGA CTA AAG GCT CAA ATA CCT TGT CCT GAT CTG TTT AGT ATA CCA TTA GAA AGA T 1141

1122 1125

N E E T R A K L **KK** **KK** L K P F F N E E G T 401

CO/17 1143 AT AAT GAA GAA ACA AGG GCA AAA TTG AAG AAG CTA AAA CCA TTC TTC AAT GAA GAA GGA A 1202

|| ||| ||| ||| ||| ||| ||| ||| ||| ||| ||| ||| ||| ||| ||| ||| ||| ||| ||| ||| |

PK/13 1142 AT AAT GAA GAA ACA AGG GCA AAA TTG AAG AAG CTA AAA CCA TTC TTC AAT GAA GAA GGA A 1201

1161 1172(aag-agg) 1179

A S L S P G M M M G M F N M L S T V L G 421

CO/17 1203 CT GCA TCT TTG TCA CCT GGG ATG ATG ATG GGA ATG TTT AAT ATG CTA TCT ACC GTG TTG G 1262

|| ||| ||| ||| ||| ||| ||| ||| ||| ||| ||| ||| ||| ||| ||| ||| ||| ||| ||| ||| |

PK/13 1202 CT GCA TCT TTG TCA CCT GGG ATG ATG ATG GGA ATG TTT AAT ATG CTA TCT ACC GTG TTG G 1261

1203 1215

V A A L G I K N I G N K E Y L W D G L Q 441

CO/17 1263 GA GTA GCT GCA CTA GGT ATC AAG AAC ATT GGA AAC AAA GAA TAC CTA TGG GAT GGA CTG C 1322

|| ||| ||| ||| ||| ||| ||| ||| ||| ||| ||| ||| ||| ||| ||| ||| ||| ||| ||| ||| |

PK/13 1262 GA GTA GCT GCA CTA GGT ATC AAG AAC ATT GGA AAC AAA GAA TAC CTA TGG GAT GGA CTG C 1321

1269 1306(cta-tta)

S S D D F **A** L F V N A K D E E T C M E G 461

CO/17 1323 AA TCT TCT GAT GAT TTT GCT CTA TTT GTT AAT GCA AAG GAT GAA GAA ACA TGT ATG GAA G 1382

|| ||| ||| ||| ||| ||| ||| ||| ||| ||| ||| ||| ||| ||| ||| ||| ||| ||| ||| ||| |

PK/13 1322 AA TCT TCT GAT GAT TTT GCT CTA TTT GTT AAT GCA AAG GAT GAA GAA ACA TGT ATG GAA G 1381

1329 1342(cta-tta) 1359

I N D F Y R T C K L L G **I** N M S K K K S 481

CO/17 1383 GA ATA AAC GAC TTT TAC CGA ACA TGT AAA TTA TTG GGA ATA AAC ATG AGC AAA AAG AAA A 1442

|| ||| ||| ||| ||| ||| ||| ||| ||| ||| ||| ||| ||| ||| ||| ||| ||| ||| ||| ||| |

PK/13 1382 GA ATA AAC GAC TTT TAC CGA ACA TGT AAA TTA TTG GGA ATA AAC ATG AGC AAA AAG AAA A 1441

1383 1392

Y C N E T G M F E F T S M F Y R D G F V 501

CO/17 1443 GT TAC TGT AAT GAG ACT GGA ATG TTT GAA TTT ACA AGC ATG TTC TAC AGA GAT GGA TTT G 1502

|| ||| ||| ||| ||| ||| ||| ||| ||| ||| ||| ||| ||| ||| ||| ||| ||| ||| ||| ||| |

PK/13 1442 GT TAC TGT AAT GAG ACT GGA ATG TTT GAA TTT ACA AGC ATG TTC TAC AGA GAT GGA TTT G 1501

1452 1488

S N F A M E L P S F G V A G V N E S A D 521

CO/17 1503 TA TCT AAT TTT GCA ATG GAA CTC CCT TCG TTT GGG GTT GCT GGA GTA AAT GAA TCA GCA G 1562

|| ||| ||| ||| ||| ||| ||| ||| ||| ||| ||| ||| ||| ||| ||| ||| ||| ||| ||| ||| |

PK/13 1502 TA TCT AAT TTT GCA ATG GAA CTC CCT TCG TTT GGG GTT GCT GGA GTA AAT GAA TCA GCA G 1561

1509 1522(ctc-att) 1537

M A I G M T I I K N N M I N N G M G P A 541

CO/17 1563 AT ATG GCA ATA GGA ATG ACA ATA ATA AAG AAC AAC ATG ATC AAC AAT GGA ATG GGT CCA G 1622

|| ||| ||| ||| ||| ||| ||| ||| ||| ||| ||| ||| ||| ||| ||| ||| ||| ||| ||| ||| |

PK/13 1562 AT ATG GCA ATA GGA ATG ACA ATA ATA AAG AAC AAC ATG ATC AAC AAT GGA ATG GGT CCA G 1621

1596 1621

T A Q T A I Q L F I A D Y R Y T Y K C H 561

CO/17 1623 CA ACA GCA CAA ACA GCC ATA CAG CTA TTC ATA GCT GAT TAT AGA TAC ACC TAC AAA TGC C 1682

|| ||| ||| ||| ||| ||| ||| ||| ||| ||| ||| ||| ||| ||| ||| ||| ||| ||| ||| ||| |

PK/13 1622 CA ACA GCA CAA ACA GCC ATA CAG CTA TTC ATA GCT GAT TAT AGA TAC ACC TAC AAA TGC C 1681

1644 1645(cta-ttg) 1671

R G D S K V E G K R M K I I K E L W E N 581

CO/17 1683 AC AGG GGA GAT TCC AAA GTA GAA GGA AAG AGA ATG AAA ATC ATA AAG GAG TTA TGG GAA A 1742

|| ||| ||| ||| ||| ||| ||| ||| ||| ||| ||| ||| ||| ||| ||| ||| ||| ||| ||| ||| |

PK/13 1682 AC AGG GGA GAT TCC AAA GTA GAA GGA AAG AGA ATG AAA ATC ATA AAG GAG TTA TGG GAA A 1741

1686 1732(tta-cta)

T K G R D G L L V **A** D G G P N I Y N L R 601

CO/17 1743 AC ACT AAA GGA AGA GAT GGT CTA TTA GTA GCA GAT GGT GGG CCC AAC ATT TAC AAT TTG A 1802

|| ||| ||| ||| ||| ||| ||| ||| ||| ||| ||| ||| ||| ||| ||| ||| ||| ||| ||| ||| |

PK/13 1742 AC ACT AAA GGA AGA GAT GGT CTA TTA GTA GCA GAT GGT GGG CCC AAC ATT TAC AAT TTG A 1801

1764

N L H I P E I V L K Y N L M D P E Y K G 621

CO/17 1803 GA AAC TTG CAT ATC CCA GAA ATA GTA TTG AAG TAT AAT CTA ATG GAC CCT GAA TAC AAA G 1862

|| ||| ||| ||| ||| ||| ||| ||| ||| ||| ||| ||| ||| ||| ||| ||| ||| ||| ||| ||| |

PK/13 1802 GA AAC TTG CAT ATC CCA GAA ATA GTA TTG AAG TAT AAT CTA ATG GAC CCT GAA TAC AAA G 1861

1809 1839

R L L H P Q N P F V G H L S I E G I K E 641

CO/17 1863 GG CGG TTA CTT CAT CCT CAA AAT CCC TTT GTG GGA CAT TTG TCT ATC GAG GGC ATC AAA G 1922

|| ||| ||| ||| ||| ||| ||| ||| ||| ||| ||| ||| ||| ||| ||| || ||| ||| ||| ||| |

PK/13 1862 GG CGG TTA CTT CAT CCT CAA AAT CCC TTT GTG GGA CAT TTG TCT ATT GAG GGC ATC AAA G 1921

1875 1911

A D I T P A H G P V **KK** K M D***** Y D A V S G 661

CO/17 1923 AG GCA GAC ATA ACT CCA GCA CAT GGT CCA GTA AAG AAA ATG GAC TAC GAT GCG GTG TCT G 1982

|| ||| ||| ||| ||| ||| ||| ||| ||| ||| ||| ||| ||| ||| ||| ||| ||| || ||| ||| |

PK/13 1922 AG GCA GAC ATA ACT CCA GCA CAT GGT CCA GTA AAG AAA ATG GAC TAC GAT GCA GTG TCT G 1981

1923 1955(aag-agg) 1974

T H S W R T K R N R S I L N T D Q R N M 681

CO/17 1983 GA ACT CAT AGT TGG AGA ACC AAA AGA AAC AGA TCT ATA CTA AAC ACT GAT CAG AGG AAC A 2042

|| ||| ||| ||| ||| ||| ||| ||| || ||| ||| ||| ||| ||| || ||| ||| ||| ||| ||| |

PK/13 1982 GA ACT CAT AGT TGG AGA ACC AAA AGG AAC AGA TCT ATA CTA AAT ACT GAT CAG AGG AAC A 2041

2007 2025

I L E E Q C Y A K C C N L F E A C F N S 701

CO/17 2043 TG ATT CTT GAG GAG CAA TGC TAC GCT AAA TGT TGC AAC CTA TTT GAG GCC TGT TTT AAC A 2102

|| ||| ||| || || ||| ||| ||| ||| ||| ||| ||| || || ||| ||| ||| ||| ||| ||| |

PK/13 2042 TG ATT CTT GAA GAA CAA TGC TAC GCT AAA TGT TGC AAT CTT TTT GAG GCC TGT TTT AAC A 2101

2052 2082

A S Y R K P V G Q H S M L E A M A H R L 721

CO/17 2103 GT GCA TCA TAC AGG AAG CCA GTG GGT CAA CAT AGC ATG CTT GAG GCT ATG GCC CAC AGA T 2162

|| ||| ||| ||| ||| || ||| || || ||| ||| ||| ||| ||| ||| ||| ||| ||| || ||| |

PK/13 2102 GT GCA TCA TAC AGG AAA CCA GTA GGG CAA CAT AGC ATG CTT GAG GCT ATG GCC CAT AGA T 2161

2118 2157

R M D A R L D Y E S G R M S K D D F E K 741

CO/17 2163 TA AGA ATG GAT GCA CGA TTA GAT TAT GAA TCA GGG AGA ATG TCA AAA GAT GAT TTT GAG A 2222

|| ||| ||| ||| ||| ||| || ||| ||| ||| ||| || ||| ||| ||| || ||| ||| ||| || |

PK/13 2162 TA AGA ATG GAT GCA CGA CTA GAT TAT GAA TCA GGA AGA ATG TCA AAG GAT GAT TTT GAA A 2221

2179(tta-cta) 2220

A M **A** H L G E I G Y I 752

CO/17 2223 AA GCA ATG GCT CAC CTT GGT GAG ATT GGG TAC ATA TAA 2260

|| ||| ||| ||| ||| ||| ||| ||| ||| ||| ||| | | |||

PK/13 2222 AA GCA ATG GCT CAC CTT GGT GAG ATT GGG TAC ACA TAA 2259

2255(ata-aca)

Protein numbering is shown on the right side for the full length PB1 ORF.

Residues highlighted in **bold** were identified in Yang et al., (2012) Table 2 as amino acid substitutions in multiple genotypes, 4/7 PB1 were subject to mutation in this analysis.

Residues highlighted in **bold** were identified in Virk et al., (2020) supplementary information as truck amino acid substitutions on phylogenetic trees, 5/11 PB1 were subject to mutation in this analysis. Amino acid positions identified as positively selected sites are marked with an asterisk *****

**PA**

M D T F I T R N F Q T T I I Q K A K N T M 21

CO/17 17 TG GAT ACT TTT ATT ACA AGA AAC TTC CAG ACT ACA ATA ATA CAA AAG GCC AAA AAC ACA A 76

|| ||| ||| ||| ||| ||| ||| ||| ||| ||| ||| ||| ||| ||| ||| ||| ||| ||| ||| ||| |

PK/13 2 TG GAT ACT TTT ATT ACA AGA AAC TTC CAG ACT ACA ATA ATA CAA AAG GCC AAA AAC ACA A 61

A E F S E D P E L Q P A M L F N I C V H 41

CO/17 77 TG GCA GAA TTT AGT GAA GAT CCT GAA TTG CAA CCA GCA ATG CTA TTC AAT ATC TGC GTC C 136

|| ||| ||| ||| ||| ||| ||| ||| ||| ||| ||| ||| ||| ||| ||| ||| || ||| || ||| |

PK/13 62 TG GCA GAA TTT AGT GAA GAT CCT GAA TTG CAA CCA GCA ATG CTA TTC AAC ATC TGT GTC C 121

L E V C Y V I S D M N F L D E E G K A Y 61

CO/17 137 AT CTA GAG GTT TGC TAT GTA ATA AGT GAC ATG AAT TTT CTT GAC GAA GAA GGA AAA GCA T 196

|| ||| ||| ||| ||| ||| ||| ||| ||| ||| ||| ||| ||| ||| ||| ||| ||| ||| ||| ||| |

PK/13 122 AT CTA GAG GTT TGC TAT GTA ATA AGT GAC ATG AAT TTT CTT GAC GAA GAA GGA AAA GCA T 180

T A L E G Q G K E Q N L R P Q Y E V I E 81

CO/17 197 AT ACA GCA TTA GAA GGA CAA GGG AAA GAA CAA AAC TTG AGA CCA CAA TAT GAA GTA ATT G 256

|| ||| ||| ||| ||| ||| ||| ||| ||| ||| ||| || ||| ||| ||| ||| ||| ||| ||| ||| |

PK/13 182 AT ACA GCA TTA GAA GGA CAA GGG AAA GAA CAA AAT TTG AGA CCA CAA TAT GAA GTA ATT G 240

G M P R T I A W M V Q R S L A Q E H G I 101

CO/17 257 AG GGA ATG CCA AGA ACC ATA GCA TGG ATG GTC CAG AGA TCC TTA GCT CAA GAG CAT GGA A 316

|| ||| ||| ||| ||| ||| ||| ||| ||| ||| ||| || ||| ||| ||| ||| ||| ||| ||| || |

PK/13 242 AG GGA ATG CCA AGA ACC ATA GCA TGG ATG GTC CAA AGA TCC TTA GCT CAA GAG CAT GGG A 300

E T P K Y L A D L F D Y K T K R F I E V 121

CO/17 317 TA GAG ACT CCC AAG TAT CTG GCT GAT TTG TTT GAT TAC AAA ACC AAA AGA TTT ATA GAA G 376

|| ||| ||| ||| ||| ||| ||| ||| ||| ||| ||| ||| || || ||| || ||| ||| ||| ||| |

PK/13 302 TA GAG ACT CCC AAG TAT CTG GCT GAT TTG TTT GAT TAT AAG ACC AAG AGA TTT ATA GAA G 360

G I T K G L A D D Y F W K K K E K L G N 141

CO/17 377 TT GGA ATA ACA AAG GGA TTG GCT GAT GAT TAC TTT TGG AAA AAG AAA GAA AAA TTG GGA A 436

|| ||| ||| ||| || ||| ||| ||| ||| ||| ||| ||| ||| ||| ||| ||| || || || ||| |

PK/13 362 TT GGA ATA ACA AAA GGA TTG GCT GAT GAT TAC TTT TGG AAA AAG AAA GAG AAG CTG GGA A 420

S M E L M I F S Y N Q D Y S L S N E S S 161

CO/17 437 AT AGC ATG GAA CTG ATG ATA TTC AGC TAC AAT CAA GAC TAC TCG TTA AGT AAT GAA TCC T 496

|| ||| ||| ||| || ||| ||| ||| ||| ||| ||| ||| ||| || ||| ||| ||| ||| ||| ||| |

PK/13 422 AT AGC ATG GAA CTA ATG ATA TTC AGC TAC AAT CAA GAC TAT TCG TTA AGT AAT GAA TCC T 480

L D E E G K G R V L S R L T E L Q A E L 181

CO/17 497 CA TTG GAT GAG GAA GGG AAA GGG AGA GTG CTA AGC AGA CTC ACA GAA CTT CAG GCT GAA T 556

|| ||| ||| ||| ||| ||| ||| ||| ||| ||| ||| ||| || ||| ||| ||| ||| || ||| ||| |

PK/13 482 CA TTG GAT GAG GAA GGG AAA GGG AGA GTG CTA AGC AGG CTC ACA GAA CTT CAA GCT GAA T 540

S L K N L W Q V L I G E E D V E K G I D 201

CO/17 557 TA AGT CTG AAA AAT TTA TGG CAA GTT CTC ATA GGA GAA GAA GAT GTT GAA AAG GGA ATT G 616

|| ||| ||| ||| || ||| ||| ||| ||| ||| ||| ||| ||| ||| ||| ||| ||| ||| ||| ||| |

PK/13 542 TA AGT CTG AAA AAC TTA TGG CAA GTT CTC ATA GGA GAA GAA GAT GTT GAA AAG GGA ATT G 600

F **K** L G Q T I S R L R D I S V P A G F S 221

CO/17 617 AT TTT AAA CTT GGA CAG ACA ATA TCT AGA CTA AGG GAT ATA TCT GTT CCA GCC GGT TTC T 676

| ||| | | ||| ||| || ||| ||| ||| ||| ||| ||| ||| ||| ||| ||| || || ||| ||| |

PK/13 601 AC TTT AGA CTT GGA CAA ACA ATA TCT AGA CTA AGG GAT ATA TCT GTT CCT GCT GGT TTC T 660

N F E G M R S Y I D N I D P K G A I E R 241

CO/17 677 CC AAT TTT GAA GGA ATG AGG AGC TAC ATA GAC AAT ATA GAC CCA AAA GGA GCA ATA GAG A 736

|| ||| ||| ||| ||| ||| ||| ||| ||| ||| ||| ||| ||| ||| || ||| ||| ||| ||| ||| |

PK/13 661 CC AAT TTT GAA GGA ATG AGG AGC TAC ATA GAC AAT ATA GAC CCG AAA GGA GCA ATA GAG A 720

N L A R M S P L V S V T P K K L **T** W E D 261

CO/17 737 GA AAT CTA GCA AGG ATG TCT CCC TTA GTA TCA GTC ACA CCT AAA AAG TTA ACA TGG GAG G 796

|| || ||| ||| ||| ||| ||| ||| ||| ||| ||| ||| ||| ||| ||| ||| || | | ||| ||| |

PK/13 721 GA AAC CTA GCA AGG ATG TCT CCC TTA GTA TCA GTC ACA CCT AAA AAG TTG AAA TGG GAG G 780

L R P I G P H I Y D H E L P E V P Y N A 281

CO/17 797 AC CTA AGA CCA ATA GGG CCT CAC ATT TAC GAC CAT GAG CTA CCA GAA GTT CCA TAT AAT G 856

|| ||| ||| ||| ||| ||| ||| ||| ||| ||| ||| ||| ||| ||| ||| ||| ||| ||| ||| ||| |

PK/13 781 AC CTA AGA CCA ATA GGG CCT CAC ATT TAC GAC CAT GAG CTA CCA GAA GTT CCA TAT AAT G 840

F L L M S D E L G L A N **M** T E G K S K K 301

CO/17 857 CC TTT CTT CTA ATG TCT GAT GAA CTA GGA TTG GCC AAT ATG ACT GAG GGA AAG TCC AAA A 916

|| ||| ||| || ||| ||| ||| ||| || || || ||| ||| ||| || ||| ||| ||| ||| ||| |

PK/13 841 CC TTT CTT TTA ATG TCT GAT GAA CTG GGG CTG GCC AAT ATG ACG GAG GGA AAG TCC AAA A 900

P K T L A K E C L E K Y S T L R D Q T D 321

CO/17 917 AA CCG AAG ACA TTA GCC AAA GAA TGT CTA GAA AAG TAC TCA ACA CTA CGG GAT CAA ACT G 976

|| || ||| ||| ||| ||| ||| ||| ||| ||| ||| ||| ||| ||| ||| ||| ||| ||| || ||| |

PK/13 901 AA CCA AAG ACA TTA GCC AAA GAA TGT CTA GAA AAG TAC TCA ACA CTA CGG GAT CAG ACT G 960

P I L I **M** K S E K A N E N F L W K L W R 341

CO/17 977 AC CCA ATA TTA ATA ATG AAA AGC GAG AAA GCT AAC GAA AAT TTC TTA TGG AAG CTT TGG A 1036

|| ||| ||| ||| ||| ||| ||| ||| || ||| ||| ||| ||| ||| ||| ||| ||| || ||| ||| |

PK/13 961 AC CCA ATA TTA ATA ATG AAA AGC GAA AAA GCT AAC GAA AAT TTC TTA TGG AAA CTT TGG A 1020

D C V N T I S N E E **A** S N E L Q K T N Y 361

CO/17 1037 GA GAC TGT GTA AAT ACA ATA AGT AAT GAG GAA GCG AGT AAC GAG TTA CAG AAA ACC AAT T 1096

|| ||| ||| ||| ||| ||| ||| ||| ||| ||| ||| | ||| ||| ||| ||| ||| ||| ||| ||| |

PK/13 1021 GA GAC TGT GTA AAT ACA ATA AGT AAT GAG GAA ATG AGT AAC GAG TTA CAG AAA ACC AAT T 1080

A K W A T G D G L T Y Q K I M K E **V** A I 381

CO/17 1097 AT GCC AAA TGG GCC ACA GGG GAT GGA TTA ACA TAC CAG AAA ATA ATG AAA GAA GTA GCA A 1156

|| ||| || ||| ||| ||| ||| ||| ||| ||| ||| ||| ||| ||| ||| ||| ||| ||| ||| ||| |

PK/13 1081 AT GCC AAG TGG GCC ACA GGG GAT GGA TTA ACA TAC CAG AAA ATA ATG AAA GAA GTA GCA A 1140

D D E T M C Q E E P K I P N K C R V A A 401

CO/17 1157 TA GAT GAC GAA ACA ATG TGC CAA GAA GAG CCT AAA ATC CCT AAC AAA TGT AGA GTG GCT G 1216

|| ||| ||| ||| ||| ||| ||| ||| ||| ||| ||| ||| ||| ||| ||| || ||| ||| ||| ||| |

PK/13 1141 TA GAT GAC GAA ACA ATG TGC CAA GAA GAG CCT AAA ATC CCT AAC AAG TGT AGA GTG GCT G 1200

W V Q T E M N L L S T L T S K R A L D L 421

CO/17 1217 CT TGG GTT CAA ACA GAG ATG AAT CTA TTG AGC ACT CTG ACA AGT AAA AGA GCT CTG GAC C 1276

|| ||| ||| ||| ||| ||| ||| ||| ||| ||| ||| ||| ||| ||| ||| ||| ||| ||| ||| ||| |

PK/13 1201 CT TGG GTT CAA ACA GAG ATG AAT CTA TTG AGC ACT CTG ACA AGT AAA AGA GCT CTG GAC C 1260

P E I G P D I A P V E H V G S E R R K Y 441

CO/17 1277 TA CCA GAA ATA GGG CCA GAC ATA GCA CCC GTA GAG CAT GTA GGA AGT GAA AGA AGG AAA T 1336

|| ||| ||| ||| ||| ||| ||| ||| ||| ||| ||| ||| ||| ||| ||| ||| ||| ||| ||| ||| |

PK/13 1261 TA CCA GAA ATA GGG CCA GAC ATA GCA CCC GTA GAG CAT GTA GGA AGT GAA AGA AGG AAA T 1320

F V N E I N Y C K A S T V M M K Y V L F 461

CO/17 1337 AC TTT GTC AAC GAA ATC AAC TAC TGT AAG GCC TCT ACA GTT ATG ATG AAG TAT GTG CTT T 1396

|| ||| || ||| ||| ||| ||| ||| ||| ||| ||| ||| ||| ||| ||| ||| ||| ||| ||| ||| |

PK/13 1321 AC TTT GTT AAC GAA ATC AAC TAC TGT AAG GCC TCT ACA GTT ATG ATG AAG TAT GTG CTT T 1380

H T S L L N E S N A S M G K Y K V I P I 481

CO/17 1397 TT CAT ACT TCA TTG TTG AAT GAA AGC AAT GCC AGC ATG GGA AAA TAC AAA GTG ATA CCA A 1456

|| ||| ||| ||| ||| ||| ||| ||| ||| ||| ||| ||| ||| ||| ||| ||| ||| || ||| ||| |

PK/13 1381 TT CAT ACT TCA TTG TTG AAT GAA AGC AAT GCC AGC ATG GGA AAA TAC AAA GTA ATA CCA A 1440

T N R **VV** V N E K G E S F D M L Y G L A V 501

CO/17 1457 TA ACC AAC AGA GTA GTA AAT GAA AAA GGA GAA AGT TTC GAC ATG CTT TAC GGT TTG GCG G 1516

|| ||| ||| ||| || ||| ||| ||| ||| ||| ||| ||| ||| ||| ||| ||| || ||| || ||| |

PK/13 1441 TA ACC AAC AGA ATA GTA AAT GAA AAA GGA GAA AGT TTC GAC ATG CTT TAT GGT CTG GCG G 1500

K G Q S H L R G D T D V V T V V T F E F 521

CO/17 1517 TT AAA GGA CAA TCT CAT CTG AGG GGA GAT ACA GAT GTT GTA ACA GTT GTA ACT TTT GAA T 1576

|| ||| ||| ||| ||| ||| ||| ||| || ||| || ||| ||| ||| ||| ||| ||| ||| ||| ||| |

PK/13 1501 TT AAA GGA CAA TCT CAT CTG AGG GGG GAT ACT GAT GTT GTA ACA GTT GTA ACT TTT GAA T 1560

S S T D P R V D **SS** G K W P K Y T V F R I 541

CO/17 1577 TT AGT AGT ACA GAT CCC AGA GTG GAC TCA GGA AAG TGG CCA AAA TAT ACT GTG TTT AGG A 1636

|| ||| ||| ||| ||| ||| ||| ||| ||| ||| ||| ||| ||| ||| ||| ||| ||| ||| ||| ||| |

PK/13 1561 TT AGT AGT ACA GAT CCC AGA GTG GAC TCA GGA AAG TGG CCA AAA TAT ACT GTG TTT AGG A 1620

G S L F V **S** G R E K S V Y L Y C R V N G 561

CO/17 1637 TT GGC TCC CTA TTT GTG AGT GGG AGG GAA AAA TCT GTG TAC TTG TAC TGC AGA GTG AAT G 1696

|| ||| ||| ||| ||| ||| ||| || ||| ||| ||| ||| ||| ||| | || ||| ||| ||| ||| |

PK/13 1621 TT GGC TCC CTA TTT GTG AGT GGA AGG GAA AAA TCT GTG TAC CTA TAT TGC AGA GTG AAT G 1680

T N K I Q M K W G M E A***** R R C L L Q S M 581

CO/17 1697 GC ACA AAT AAG ATC CAA ATG AAA TGG GGA ATG GAA GCT AGA AGA TGT TTG CTT CAA TCA A 1756

|| ||| ||| ||| ||| ||| ||| ||| ||| ||| ||| ||| ||| ||| ||| ||| || ||| ||| ||| |

PK/13 1681 GC ACA AAT AAG ATC CAA ATG AAA TGG GGA ATG GAA GCT AGA AGA TGT CTG CTT CAA TCA A 1740

Q Q M E A I V E Q E S S I Q G Y D M T K 601

CO/17 1757 TG CAA CAA ATG GAG GCA ATT GTT GAA CAG GAA TCA TCA ATA CAA GGA TAT GAC ATG ACC A 1816

|| ||| ||| ||| || ||| ||| ||| ||| || ||| || || ||| ||| ||| || ||| ||| ||| |

PK/13 1741 TG CAA CAA ATG GAA GCA ATT GTT GAA CAA GAA TCC TCG ATA CAA GGA TAC GAC ATG ACC A 1800

A C F K G D R **V** N S P K T F S **II** G T***** Q E 621

CO/17 1817 AA GCC TGT TTC AAG GGA GAC AGA GTA AAT AGC CCC AAA ACT TTC AGT ATT GGA ACT CAA G 1876

|| || ||| ||| ||| || ||| ||| ||| ||| ||| ||| ||| ||| ||| ||| ||| ||| ||| ||| |

PK/13 1801 AA GCT TGT TTC AAG GGG GAC AGA GTA AAT AGC CCC AAA ACT TTC AGT ATT GGA ACT CAA G 1860

G K L V K G S F G K A L R V I F T K C L 641

CO/17 1877 AA GGA AAA CTA GTA AAA GGA TCC TTT GGA AAA GCA CTA AGA GTA ATA TTT ACT AAA TGC T 1936

|| || ||| ||| ||| ||| ||| ||| ||| ||| ||| ||| ||| ||| ||| ||| ||| ||| ||| || |

PK/13 1861 AA GGG AAA CTA GTA AAA GGA TCC TTT GGA AAA GCA CTA AGA GTA ATA TTT ACT AAA TGT T 1920

M H Y V F G N A Q L E G F S A E S R R L 661

CO/17 1937 TG ATG CAC TAT GTA TTT GGA AAT GCC CAA TTG GAG GGG TTT AGT GCC GAG TCT AGG AGA C 1996

|| ||| ||| ||| ||| ||| ||| ||| ||| || ||| ||| || || ||| ||| ||| ||| ||| ||| |

PK/13 1921 TG ATG CAC TAT GTA TTT GGA AAT GCC CAG TTG GAG GGA TTC AGT GCC GAG TCT AGG AGA C 1980

L L L I Q A L K D R K G P W V F D L E G 681

CO/17 1997 TT CTA TTG TTG ATT CAA GCA TTA AAG GAC AGA AAG GGT CCT TGG GTG TTC GAC TTA GAG G 2056

|| ||| || ||| ||| ||| ||| ||| ||| ||| ||| ||| || ||| ||| ||| ||| ||| ||| ||| |

PK/13 1981 TT CTA CTG TTG ATT CAA GCA TTA AAG GAC AGA AAG GGC CCT TGG GTG TTC GAC TTA GAG G 2040

M Y S G **I** E E C I S N N P W V I Q S V Y 701

CO/17 2057 GA ATG TAT TCT GGA ATA GAA GAA TGT ATT AGC AAC AAC CCT TGG GTG ATA CAG AGT GTA T 2116

|| ||| ||| ||| ||| ||| ||| ||| ||| || || ||| ||| ||| ||| || ||| ||| ||| | | |

PK/13 2041 GA ATG TAT TCT GGA ATA GAA GAA TGT ATC AGT AAC AAC CCT TGG GTA ATA CAG AGT GCA T 2100

W F N E W L G **F** E K E G S K V L E S V D 721

CO/17 2117 AC TGG TTC AAT GAA TGG TTG GGC TTT GAA AAG GAG GGG AGT AAA GTG TTG GAA TCA GTG G 2176

|| ||| ||| ||| ||| ||| ||| || ||| ||| ||| ||| || ||| ||| || || ||| ||| || |

PK/13 2101 AC TGG TTC AAT GAA TGG TTG GGT TTT GAA AAG GAG GGA AGT AAA GTA TTA GAA TCA GTA G 2160

E **V** M D E

CO/17 2177 AT GAA GTA ATG GAT GAA TAA 2196

|| ||| || ||| ||| ||| |||

PK/13 2161 AT GAA ATA ATG GAT GAA TAA 2180

Protein numbering is shown on the right side for the full length PA ORF.

Residues highlighted in **bold** were identified in Chen and Holmes (2008) Table 2 as positively selected amino acid positions, 1/1 PA were subject to mutation in this analysis.

Residues highlighted in **bold** were identified in Yang et al., (2012) Table 2 as amino acid substitutions in multiple genotypes, 1/3 PA were subject to mutation in this analysis.

Residues highlighted in **bold** were identified in Virk et al., (2020) supplementary information as truck amino acid substitutions on phylogenetic trees, 6/13 PA were subject to mutation in this analysis. Amino acid positions identified as positively selected sites are marked with an asterisk *****

**HA**

HA 1 M K A I I V L L M V V T S S 14

CO/17 1 ATTTTCTAATATCCACAAA ATG AAG GCA ATA ATT GTA CTA CTC ATG GTA GTA ACA TCC AG 60

||||||||||||||||||| ||| ||| ||| ||| ||| ||| ||| ||| ||| ||| ||| ||| ||| |

PK/13 1 ATTTTCTAATATCCACAAA ATG AAG GCA ATA ATT GTA CTA CTC ATG GTA GTA ACA TCC AA 60

A D R I***** C T G***** I T S S N S P H V***** V K T A 34**/19**

CO/17 61 T GCA GAT CGA ATC TGC ACT GGG ATA ACA TCG TCA AAC TCA CCA CAT GTC GTC AAA ACT GC 120

||| ||| ||| ||| ||| ||| ||| ||| ||| || ||| ||| ||| || ||| || ||| ||| || ||

PK/13 61 C GCA GAT CGA ATC TGC ACT GGG ATA ACA TCT TCA AAC TCA CCT CAT GTG GTC AAA ACA GC 120

T Q G E V N V T G***** V I P L T T T P T K S 54**/39**

CO/17 121 T ACT CAA GGG GAG GTC AAT GTG ACC GGT GTA ATA CCA CTG ACA ACA ACA CCC ACC AAA TC 180

| ||| ||| ||| ||| ||| ||| ||| || || || ||| ||| ||| ||| ||| ||| || || ||| ||

PK/13 121 T ACT CAA GGG GAG GTC AAT GTG ACT GGC GTG ATA CCA CTG ACA ACA ACA CCA ACA AAA TC 180

H***** F A N L K G T **EE** T R***** G K L C P **K** C **L** N 74**/59**

CO/17 181 T CAT TTT GCA AAT CTC AAA GGA ACA GAA ACC AGG GGG AAA CTA TGC CCA AAA TGC CTC AA 240

| || ||| ||| ||| ||| ||| ||| ||| ||| || ||| ||| ||| ||| || | || ||| ||

PK/13 181 T TAT TTT GCA AAT CTC AAA GGA ACA AGG ACC AGA GGG AAA CTA TGC CCG GAC TGT CTC AA 240

C T D L D V***** A L G R P K***** C T G***** K **I** P S A 94**/79**

CO/17 241 C TGC ACA GAT CTG GAT GTA GCC TTG GGC AGA CCA AAA TGC ACA GGG AAA ATA CCC TCT GC 300

| || ||| ||| ||| ||| || ||| ||| ||| || ||| | || ||| | | | || ||| ||

PK/13 241 C TGT ACA GAT CTG GAT GTG GCC TTG GGC AGG CCA ATG TGT GTG GGG ACC ACA CCT TCT GC 300

R V S I L H***** E V R P V T S G C F P I M H 114**/99**

CO/17 301 A AGG GTT TCA ATA CTC CAT GAA GTC AGA CCT GTT ACA TCT GGG TGC TTT CCT ATA ATG CA 360

| | | ||| ||| ||| ||| || ||| ||| ||| ||| ||| || ||| ||| ||| ||| ||| ||| ||

PK/13 301 T AAA GCT TCA ATA CTC CAT GAG GTC AGA CCT GTT ACA TCC GGG TGC TTT CCT ATA ATG CA 360

D R T K I R***** Q L **PP*** N L L R***** G Y E **HH** **V** R***** L 134**/119**

CO/17 361 C GAT AGA ACA AAA ATT AGA CAG CTG CCT AAC CTT CTC CGA GGA TAC GAA CAT GTC AGG TT 420

| || ||| ||| ||| || || || || || || ||| ||| || ||| || ||| | || ||| ||

PK/13 361 C GAC AGA ACA AAA ATC AGG CAA CTA CCC AAT CTT CTC AGA GGA TAT GAA AAG ATC AGG TT 420

S T H***** N V I N A E **G*** A P G **G** P Y **K** I G T 154**/139**

CO/17 421 A TCA ACT CAC AAC GTT ATC AAT GCA GAA GGT GCA CCA GGA GGA CCC TAC AAA ATT GGA AC 480

| ||| || || ||| ||| ||| || ||| ||| ||| ||| ||| ||| ||| ||| | | || ||| ||

PK/13 421 A TCA ACC CAA AAC GTT ATC GAT GCA GAA AAA GCA CCA GGA GGA CCC TAC AGA CTT GGA AC 480

S G S C P N **I*** T N **G** **NN** G***** F F***** A T M A W A 174**/159**

CO/17 481 C TCA GGG TCT TGC CCT AAC ATT ACC AAT GGA AAC GGA TTC TTC GCA ACA ATG GCT TGG GC 540

| ||| || ||| ||| ||| ||| | ||| | | | | | ||| || || ||| ||| ||| ||| ||| ||

PK/13 481 C TCA GGA TCT TGC CCT AAC GCT ACC AGT AAA ATC GGA TTT TTT GCA ACA ATG GCT TGG GC 540

V P D K **NN** **K** **T** A T N P **L** T I E V P Y V 193**/178**

CO/17 541 C GTC CCA --- GAC AAA AAC AAA ACA GCA ACA AAT CCA TTA ACA ATA GAA GTA CCA TAC GT 597

||| ||| ||| || || ||| | ||| || || ||| || ||| | ||| ||| ||| ||| |

PK/13 541 T GTC CCA AAG GAC AAC TAC AAA AAT GCA ACG AAC CCA CTA ACA GTG GAA GTA CCA TAC AT 600

C **T** E G E D Q I T V W G F H S***** D***** N***** E***** **TT** Q 213**/198**

CO/17 598 T TGT ACA GAA GGA GAA GAC CAA ATT ACC GTT TGG GGG TTC CAC TCT GAC A**T**C GAG ACC CA 657

| ||| ||| ||| || ||| ||| ||| ||| || ||| ||| ||| ||| || || || ||| | || ||

PK/13 601 T TGT ACA GAA GGG GAA GAC CAA ATT ACT GTT TGG GGG TTC CAT TCG GAT AAC AAA RCC CA 660

M **AA*** **K*** L Y G D S K P***** **Q** K F T S S A N G V 233**/218**

CO/17 658 A ATG GCA AAG CTC TAT GGG GAC TCA AAG CCC CAG AAG TTC ACC TCA TCT GCC AAC GGA G 716

| ||| | ||| ||| || ||| ||| || || || ||| ||| ||| ||| ||| || || ||| |

PK/13 661 A ATG AAG AGC CTC TAT GGA GAC TCA AAT CCT CAA AAG TTC ACC TCA TCT GCT AAT GGA G 719

T T H Y***** V S Q I G **GG****F P***** **N** Q T***** E D G G L 253**/238**

CO/17 717 TG ACC ACA CAT TAC GTT TCA CAG ATT GGT GGC TTC CCA AAT CAA ACA GAA GAC GGA GGA C 776

| ||| || ||| || ||| || ||| ||| || | | ||| ||| || ||| ||| ||| ||| ||| ||| |

PK/13 720 TA ACC ACG CAT TAT GTT TCT CAG ATT GGC GAC TTC CCA GAT CAA ACA GAA GAC GGA GGA C 779

P Q S G R I V V D Y M **V** Q K S G K T G T 273**/258**

CO/17 777 TA CCA CAA AGT GGC AGA ATT GTT GTT GAT TAC ATG GTG CAA AAA TCT GGA AAA ACA GGA A 836

|| ||| ||| || ||| ||| ||| ||| ||| ||| ||| ||| || ||| ||| || || ||| ||| ||| |

PK/13 780 TA CCA CAA AGC GGC AGA ATT GTT GTT GAT TAC ATG ATG CAA AAA CCT GGG AAA ACA GGA A 839

I T Y **Q** R G I L L P Q K***** V W C A S G R S 293**/278**

CO/17 837 CA ATT ACC TAT CAA AGA GGT ATT TTA TTG CCT CAA AAG GTG TGG TGC GCA AGT GGC AGG A 896

|| ||| | ||| ||| || ||| || || ||| ||| ||| ||| ||| ||| ||| || ||| ||| ||| |

PK/13 840 CA ATT GTC TAT CAA AGG GGT GTT TTG TTG CCT CAA AAG GTG TGG TGC GCG AGT GGC AGG A 899

K***** V I K G S L P L I***** G E A **D** C L H***** E **K** Y 313**/298**

CO/17 897 GC AAG GTA ATA AAA GGA TCC TTG CCT TTA ATT GGA GAA GCA GAT TGC CTC CAT GAA AAA T 956

|| || ||| ||| ||| || || ||| ||| ||| ||| || ||| ||| ||| ||| || ||| ||| || |

PK/13 900 GC AAA GTA ATA AAA GGG TCA TTG CCT TTA ATT GGT GAA GCA GAT TGC CTT CAT GAA GAA T 959

G G L N K S K P Y Y T G***** **E** H A K***** A I G N 333**/318**

CO/17 957 AC GGT GGA TTA AAC AAA AGC AAG CCT TAC TAC ACA GGG GAA CAT GCA AAG GCC ATA GGA A 1016

|| ||| ||| ||| ||| ||| ||| ||| ||| ||| ||| ||| || || ||| ||| || ||| ||| ||| |

PK/13 960 AC GGT GGA TTA AAC AAA AGC AAG CCT TAC TAC ACA GGA AAA CAT GCA AAA GCC ATA GGA A 1019

C P I W V K T P***** L K L***** A N G T K Y R P P 353**/338**

CO/17 1017 AT TGC CCA ATA TGG GTG AAA ACA CCC TTG AAG CTG GCC AAT GGA ACC AAA TAT AGA CCT C 1076

|| ||| ||| ||| ||| || ||| ||| || ||| ||| || ||| ||| ||| ||| ||| ||| ||| ||| |

PK/13 1020 AT TGC CCA ATA TGG GTA AAA ACA CCT TTG AAG CTT GCC AAT GGA ACC AAA TAT AGA CCT C 1079

A K L L K E R G F F G A I A G F L E G G 373**/358**

CO/17 1077 CT GCA AAA CTA TTA AAG GAA AGG GGT TTC TTC GGA GCT ATT GCT GGT TTC TTA GAG GGA G 1136

|| ||| ||| ||| || ||| ||| ||| ||| ||| ||| ||| ||| ||| ||| ||| ||| || || ||| |

PK/13 1080 CT GCA AAA CTA TTG AAG GAA AGG GGT TTC TTC GGA GCT ATT GCT GGT TTC CTA GAA GGA G 1139

W E G M I A G W H G Y T***** S H G A H G V A 393**/378**

CO/17 1137 GA TGG GAA GGA ATG ATT GCA GGT TGG CAC GGA TAC ACA TCC CAT GGG GCA CAT GGA GTA G 1196

|| ||| ||| ||| ||| ||| ||| ||| ||| ||| ||| ||| ||| || || || ||| ||| ||| || |

PK/13 1140 GA TGG GAA GGA ATG ATT GCA GGT TGG CAC GGA TAC ACA TCT CAC GGA GCA CAT GGA GTG G 1199

***** V***** A***** A D L K S T Q E A I N K I T K N L N 413**/398**

CO/17 1197 CG GTG GCA GCT GAC CTT AAG AGC ACT CAA GAG GCC ATA AAC AAG ATA ACA AAA AAT CTC A 1256

| ||| || || ||| ||| ||| || || ||| || || ||| || ||| ||| ||| ||| ||| ||| |

PK/13 1200 CA GTG GCG GCA GAC CTT AAG AGT ACA CAA GAA GCT ATA AAT AAG ATA ACA AAA AAT CTC A 1259

***** S L S E L***** E V K N***** L Q R***** L S G A M D E L 433**/418**

CO/17 1257 AC TCT TTG AGT GAG CTG GAA GTA AAG AAT CTT CAA AGA CTA AGC GGT GCC ATG GAT GAA C 1316

| ||| ||| ||| || || ||| ||| ||| || ||| ||| ||| ||| || ||| ||| ||| ||| ||| |

PK/13 1260 AT TCT TTG AGT GAA CTA GAA GTA AAG AAC CTT CAA AGA CTA AGT GGT GCC ATG GAT GAA C 1319

H N E I L E L D***** E K V D D L R A D T I S 453**/438**

CO/17 1317 TC CAC AAC GAA ATA CTA GAA CTA GAT GAG AAA GTG GAT GAT CTC AGA GCT GAT ACA ATA A 1376

|| ||| ||| ||| ||| || || || ||| || ||| ||| ||| ||| ||| ||| ||| || || ||| |

PK/13 1320 TC CAC AAC GAA ATA CTC GAG CTG GAT GAA AAA GTG GAT GAT CTC AGA GCT GAC ACT ATA A 1379

***** S Q I E L A V L L S N E G I I N S E D E 473**/458**

CO/17 1377 GC TCA CAA ATA GAA CTC GCA GTC CTG CTT TCC AAT GAA GGA ATA ATA AAC AGC GAA GAT G 1436

|| ||| ||| ||| ||| || ||| ||| || ||| ||| || ||| ||| ||| ||| ||| || ||| || |

PK/13 1380 GC TCA CAA ATA GAA CTT GCA GTC TTG CTT TCC AAC GAA GGA ATA ATA AAC AGT GAA GAC G 1439

***** H L L A***** L E R K L K K M L G P S A V **E** I 493**/478**

CO/17 1437 AA CAT CTC TTG GCG CTT GAA AGA AAG CTG AAG AAA ATG CTG GGC CCC TCT GCT GTA GAG A 1496

| ||| || ||| || ||| || ||| || || ||| ||| ||| ||| || ||| ||| ||| ||| || |

PK/13 1440 AG CAT CTA TTG GCA CTT GAG AGA AAA CTA AAG AAA ATG CTG GGT CCC TCT GCT GTA GAC A 1499

G N G C F E T K H K C N Q T C L D K I 512**/497**

CO/17 1497 TA GGG AAT GGA TGC TTT GAA ACC AAA CAC AAG TGC AAC CAG ACC TGT CTT GAC AAA ATA 1555

|| || || ||| ||| || ||| ||| ||| ||| || ||| ||| ||| ||| || | ||| | |||

PK/13 1500 TA GGA AAC GGA TGC TTC GAA ACC AAA CAC AAA TGC AAC CAG ACC TGC TTA GAC AGG ATA 1558

**A** A G T F D A G E***** F S L P T***** F D S L N I 532**/517**

CO/17 1556 GCT GCT GGT ACC TTT GAT GCA GGA GAA TTT TCT CTC CCC ACC TTT GAT TCA CTG AAT ATT 1615

||| ||| || ||| ||| || ||| ||| ||| ||| ||| ||| ||| || ||| ||| ||| || || |||

PK/13 1559 GCT GCT GGC ACC TTT AAT GCA GGA GAA TTT TCT CTC CCC ACT TTT GAT TCA TTG AAC ATT 1618

T A A S L N D D G***** L D N***** H T I L L Y***** Y S 552**/537**

CO/17 1616 ACT GCT GCA TCT TTA AAT GAC GAT GGA TTG GAT AAT CAT ACT ATA CTG CTT TAC TAC TCA 1675

||| ||| ||| ||| ||| ||| || ||| ||| ||| ||| || ||| ||| ||| ||| || || ||| |||

PK/13 1619 ACT GCT GCA TCT TTA AAT GAT GAT GGA TTG GAT AAC CAT ACT ATA CTG CTC TAT TAC TCA 1678

T A A S S***** L A V T L M I A I F V V Y M V 572**/557**

CO/17 1676 ACT GCT GCC TCC AGT TTG GCT GTA ACA CTG ATG ATA GCT ATC TTT GTT GTT TAT ATG GTC 1735

||| ||| || || ||| ||| ||| ||| ||| | ||| || ||| || ||| ||| ||| ||| ||| ||

PK/13 1679 ACT GCT GCT TCT AGT TTG GCT GTA ACA TTA ATG CTA GCT ATT TTT ATT GTT TAT ATG GTC 1738

S R D***** N V S C S I C L *

CO/17 1736 TCC AGA GAC AAT GTT TCT TGC TCC ATT TGT CTA TAA GGAAAGTTAAGCCCTGTATTTTCC 1795

||| ||| ||| || ||| || ||| ||| || ||| ||| ||| |||| |||| ||| ||||||||||

PK/13 1739 TCC AGA GAC AAC GTT TCA TGC TCC ATC TGT CTA TAA GGAAGGTTAGGCCTTGTATTTTCC 1798

CO/17 1796 TTTATTGTAGTGCTTGGTTGCTTGTTGTCATTACAAAGAAACGTTATTGAAAAAT 1850

|||||||||||||||| |||||||| ||||||||||||||||||||||||||||

PK/13 1799 TTTATTGTAGTGCTTGTTTGCTTGTCATCATTACAAAGAAACGTTATTGAAAAAT 1853

Protein numbering is shown on the right side for the full length and signal peptide cleaved HA (**bold**).

Residues highlighted in **bold** were identified in Chen and Holmes (2008) Table 2 as positively selected amino acid positions, 7/9 HA were subject to mutation in this analysis.

Residues highlighted in **bold** were identified in Yang et al., (2012) Table 2 as amino acid substitutions in multiple genotypes, 6/7 HA were subject to mutation in this analysis.

Amino acid positions identified as positively selected sites in Yoshihara et al., (2020) are marked with an asterisk *****, 6/6 HA were subject to mutation in this analysis. Amino acid positions identified as negatively selected sites are marked with an asterisk *****, 50/65 HA were subject to mutation in this analysis.

Residues highlighted in **bold** were identified in Virk et al., (2020) as truck amino acid substitutions on phylogenetic trees, 19/23 HA were subject to mutation in this analysis. Amino acid positions identified as positively selected sites are marked with an asterisk *****

**NP**

M S N M D I D G **M** N T G T I D **K** T P E E 20

CO/17 47 ATG TCC AAC ATG GAT ATC GAC GGT ATG AAC ACT GGG ACA ATT GAC AAA ACA CCG GAA GAA 106

||| ||| ||| ||| ||| || ||| ||| || ||| ||| ||| ||| || ||| ||| ||| ||| ||| |||

PK/13 1 ATG TCC AAC ATG GAT AT**C** GAC GGT AT**G** AAC ACT GGG ACA AT**T** GAC AAA ACA CCG GAA GAA 60

I T S G T S G T T R P I I R P A T L A P 40

CO/17 107 ATA ACT TCT GGA ACC AGT GGG ACA ACC AGA CCA ATC ATT AGA CCA GCA ACC CTT GCC CCA 166

||| || ||| ||| ||| ||| ||| ||| ||| ||| ||| ||| ||| ||| ||| ||| ||| ||| ||| |||

PK/13 61 ATA ACC TCT GGA ACC AGT GGG ACA ACC AGA CCA ATC ATT AGA CCA GCA ACC CTT GCC CCA 120

P S N K R T R N P S P E R A T T S **S** E D 60

CO/17 167 CCA AGC AAC AAA CGA ACC CGT AAC CCA TCC CCG GAA AGA GCA ACC ACA AGC AGT GAA GAT 226

||| ||| ||| ||| ||| ||| ||| ||| ||| ||| ||| ||| ||| ||| ||| ||| ||| ||| ||| |||

PK/13 121 CCA AGC AAC AAA CGA ACC CGT AAC CCA TCC CCG GAA AGA GCA ACC ACA AGC AGT GAA GAT 180

D **V** G R K **A** Q K K Q T P T E I K K S V Y 80

CO/17 227 GAT GTC GGA AGG AAA GCC CAA AAG AAG CAG ACC CCG ACA GAG ATA AAG AAG AGC GTC TAC 286

||| ||| ||| ||| ||| || ||| ||| || ||| ||| ||| ||| ||| ||| ||| ||| ||| ||| |||

PK/13 181 GAT GTC GGA AGG AAA **G**CC CAA AAG AA**G** CAG ACC CCG ACA GAG ATA AAG AAG AGC GTC TAC 240

N M V V K L G E F Y N Q M M V K A G L N 100

CO/17 287 AAC ATG GTA GTG AAA TTG GGC GAA TTC TAC AAC CAG ATG ATG GTT AAA GCT GGA CTC AAT 346

||| ||| || ||| ||| || ||| ||| ||| || ||| ||| ||| ||| || ||| ||| ||| ||| |||

PK/13 241 AAC ATG GTG GTG AAA CTG GGC GAA TTC TA**C** AAC CAG ATG ATG GT**T** AAA GCT GGA CTC AAT 300

D D M E R N L I Q N A **H** A V E R I L L A 120

CO/17 347 GAT GAC ATG GAG AGA AAT CTA ATC CAA AAT GCG CAT GCC GTG GAA AGA ATT TTA TTG GCT 406

||| ||| ||| ||| ||| || ||| ||| ||| ||| ||| || || ||| ||| ||| ||| || ||| |||

PK/13 301 GAT GAC ATG GAG AGA AA**T** CTA ATC CAA AAT GCG **C**AT GCG GTG GAA AGA ATT **T**TA TTG GCT 360

A T D D K K T E F Q K K K N A R D V K E 140

CO/17 407 GCC ACT GAT GAC AAG AAA ACC GAG TTC CAG AAG AAA AAG AAT GCC AGA GAT GTC AAA GAA 466

||| ||| ||| ||| ||| ||| ||| ||| ||| ||| ||| ||| ||| ||| ||| ||| ||| ||| ||| |||

PK/13 361 GCC ACT GAT GAC AAG AAA ACC GAG TTC CAG AAG AAA AAG AAT GCC AGA GAT GTC AAA GAA 420

G K E E I D H N K T G G T F Y K M V R D 160

CO/17 467 GGG AAG GAA GAA ATA GAT CAC AAC AAA ACA GGA GGC ACC TTT TAC AAG ATG GTA AGA GAT 526

||| || ||| ||| ||| ||| ||| ||| ||| ||| ||| ||| ||| ||| ||| ||| ||| ||| ||| |||

PK/13 421 GGG AA**G** GAA GAA ATA GAT CAC AAC AAA ACA GGA GGC ACC TTT TAC AAG ATG GTA AGA GAT 480

D K T I Y F S P I R I T F L K E E V K T 180

CO/17 527 GAT AAA ACC ATC TAT TTC AGC CCT ATA AGA ATT ACC TTT TTA AAA GAA GAG GTG AAA ACA 586

|| ||| ||| ||| || ||| || ||| ||| ||| ||| ||| ||| ||| ||| ||| ||| ||| ||| |||

PK/13 481 GAC AAA ACC ATC TA**T** TTC AGT CCT ATA AGA ATT ACC TTT TTA AAA GAA GAG GTG AAA ACA 540

M Y K T T M G S D G F S G L N H I M I G 200

CO/17 587 ATG TAC AAA ACC ACC ATG GGG AGT GAT GGC TTC AGT GGA TTA AAT CAC ATA ATG ATT GGG 646

||| ||| ||| ||| ||| ||| ||| ||| ||| ||| ||| ||| ||| || ||| ||| ||| ||| ||| |||

PK/13 541 ATG TAC AAA ACC ACC ATG GGG AGT GAT GGC TTC AGT GGA **T**TA AAT CAC ATA ATG ATT GGG 600

H S Q M N D V C F Q R S K A L K R V G L 220

CO/17 647 CAT TCA CAG ATG AAT GAT GTC TGT TTC CAA AGA TCA AAG GCA CTA AAA AGA GTT GGA CTT 706

||| ||| ||| ||| ||| ||| ||| ||| ||| ||| ||| ||| ||| ||| ||| ||| ||| ||| ||| |||

PK/13 601 CAT TCA CAG ATG AAT GAT GTC TGT TTC CAA AGA TCA AAG GCA CTA AAA AGA GTT GGA CTT 660

D P S L I S T F A G S T **V** P R R S G A T 240

CO/17 707 GAT CCT TCA TTA ATC AGT ACC TTT GCG GGA AGC ACA GTC CCC AGA AGA TCA GGT GCG ACT 766

||| ||| ||| ||| ||| || ||| ||| ||| ||| ||| ||| ||| ||| ||| ||| ||| ||| ||| |||

PK/13 661 GAT CCT TCA TTA ATC AG**T** A**T**C TTT GCG GGA AGC ACA GTC CCC AGA AGA TCA GGT GCG ACT 720

G V***** A I K G G G T L V A E A I R F I G R 260

CO/17 767 GGT GTT GCA ATC AAA GGA GGT GGA ACC TTA GTG GCT GAA GCC ATT CGA TTT ATA GGA AGA 826

||| ||| ||| ||| ||| ||| || ||| || ||| ||| ||| ||| ||| ||| ||| ||| ||| ||| |||

PK/13 721 GGT GTT GCA ATC AAA GGA GG**T** GGA AC**C** TTA GTG GCT GAA GCC ATT CGA TTT ATA GGA AGA 780

A M A D R G L L R D I K A K T A Y E K I 280

CO/17 827 GCA ATG GCA GAC AGA GGG CTA TTG AGA GAC ATC AAA GCC AAG ACT GCC TAT GAA AAG ATT 886

||| ||| ||| || ||| ||| ||| ||| ||| ||| ||| ||| ||| ||| ||| ||| ||| ||| ||| |||

PK/13 781 GCA ATG GCA GA**C** AGA GGG CTA TTG AGA GAC ATC A**G**A GCC AAG ACT GCC TAT GAA AAG ATT 840

L L N L K N K C S A P Q Q K A L V D Q V 300

CO/17 887 CTT CTG AAT CTG AAG AAC AAA TGC TCT GCG CCC CAA CAA AAG GCT CTA GTT GAT CAA GTG 946

||| ||| ||| || || ||| ||| ||| ||| ||| ||| ||| ||| ||| ||| ||| ||| ||| ||| |||

PK/13 841 CTT CTG AAT CTA AA**G** AAC AAA TGC TCT GCG CCC CAA CAA AAG GCT CTA GTT GAT CAA GTG 900

I G S R N P G I A D I E D L T L L A R S 320

CO/17 947 ATC GGA AGC AGA AAT CCG GGG ATT GCA GAC ATT GAA GAT CTA ACC CTG CTT GCT CGT AGT 1006

||| ||| ||| ||| ||| ||| ||| ||| ||| ||| ||| ||| ||| ||| ||| ||| ||| ||| ||| |||

PK/13 901 ATC GGA AGC AGA AAT CCG GGG ATT GCA GAC ATT GAA GAT CTA ACC CTG CTT GCT CGT AGT 960

M V V V R P S V A S K V V L P I S I Y A***** 340

CO/17 1007 ATG GTC GTT GTT AGG CCC TCT GTG GCA AGC AAA GTG GTG CTT CCC ATA AGC ATT TAC GCC 1066

||| ||| ||| ||| ||| ||| ||| ||| ||| ||| ||| ||| ||| ||| ||| ||| ||| ||| ||| |||

PK/13 961 ATG GTC GTT GTT AGG CCC TCT GTG GCA AGC AAA GTG GTG CTT CCC ATA AGC ATT TAC GCC 1020

K I P Q L G F N V E E Y S M V G Y***** E A M 360

CO/17 1067 AAA ATA CCT CAA CTA GGG TTC AAT GTT GAA GAG TAC TCT ATG GTT GGG TAC GAA GCC ATG 1126

||| ||| || ||| ||| ||| ||| ||| ||| ||| ||| ||| ||| ||| ||| ||| || ||| ||| |||

PK/13 1021 AAA ATA CCA CAA CTA GGG TTC AAT GTT GAA GAG TAC TCT ATG GTT GGG TA**C** GAA GCC ATG 1080

A L Y N M A T P V S I L R M G D D A K D 380

CO/17 1127 GCT CTT TAC AAT ATG GCA ACA CCT GTT TCC ATA TTA AGA ATG GGG GAT GAT GCA AAG GAT 1186

||| ||| ||| ||| ||| ||| ||| ||| || ||| ||| ||| ||| ||| || ||| ||| ||| || |||

PK/13 1081 GCT CTT TAC AAT ATG GCA ACA CCT GT**T** TCC ATA TTA AGA ATG GG**G** GAT GAT GCA AA**G** GAT 1140

K S Q L F F M S C F G A A Y E D L R V L 400

CO/17 1187 AAA TCG CAA TTA TTC TTC ATG TCT TGC TTC GGA GCT GCC TAT GAA GAC CTG AGA GTT TTG 1246

||| ||| ||| ||| ||| ||| ||| ||| ||| ||| ||| ||| ||| ||| ||| ||| ||| ||| ||| |||

PK/13 1141 AAA TCG CAA TTA TTC TTC ATG TCT TGC TTC GG**G** GCT GCC TAT GAA GAC CTG AGA GTT TTG 1200

S A L T G T E F K P R S A L K C K G F H 420

CO/17 1247 TCT GCA TTA ACA GGC ACA GAA TTC AAG CCT AGA TCA GCA TTA AAA TGC AAG GGT TTC CAT 1306

||| ||| ||| ||| ||| ||| ||| ||| ||| ||| ||| ||| ||| ||| ||| ||| ||| ||| ||| |||

PK/13 1201 TCT GCA TTA ACA GGC ACA GAA TTC AAG CCT AGA TCA GCA TTA AAA TGC AAG GGT TTC CAT 1260

V P A K E Q V E G M G A A L M S I K L Q 440

CO/17 1307 GTT CCA GCA AAG GAA CAG GTA GAA GGA ATG GGA GCA GCT CTG ATG TCC ATC AAG CTC CAG 1366

||| ||| ||| ||| ||| ||| ||| ||| ||| ||| ||| ||| ||| ||| ||| ||| ||| ||| ||| |||

PK/13 1261 GTT CCA GCA AAG GAA CAG GTA GAA GGA ATG GGA GCA GCT CTG ATG TCC ATC AAG CTC CAG 1320

F W A P M T R S G G N***** E **V*** G G D G G S G 460

CO/17 1367 TTT TGG GCT CCG ATG ACC AGA TCT GGG GGA AAC GAA GTA GGT GGA GAT GGA GGG TCT GGC 1426

||| ||| ||| ||| ||| ||| ||| ||| ||| || ||| ||| | | ||| ||| || ||| ||| ||| |||

PK/13 1321 TTT TGG GCT CCG ATG ACC AGA TCT GGG GGG AAC GAA G**T**A GGT GGA GAC GGA GGG TCT GGC 1380

Q I S C S P V F A V E R P I A L S K Q A 480

CO/17 1427 CAA ATA AGC TGC AGC CCA GTG TTT GCA GTG GAG AGA CCT ATT GCT CTA AGC AAG CAA GCT 1486

||| ||| ||| ||| || ||| ||| ||| ||| ||| || ||| ||| ||| ||| ||| ||| ||| ||| |||

PK/13 1381 CAA ATA AGC TGC AG**C** CCA GTG TTT GCA GTG GA**G** AGA CCT ATT GCT CTA AGC AAG CAA GCT 1440

V R R M L S M N I E G R D A D V K G N L 500

CO/17 1487 GTA AGA AGA ATG CTG TCA ATG AAT ATT GAG GGG CGT GAT GCA GAT GTC AAA GGA AAT CTA 1546

||| ||| ||| ||| ||| ||| ||| ||| ||| ||| || ||| ||| ||| ||| ||| ||| ||| ||| |||

PK/13 1441 GTA AGA AGA ATG CTG TCA ATG AAT ATT GAG GG**G** CGT GAT GCA GAT GTC AAA GGA AAT CTA 1500

L K M M N D S M A K K T **S** G N A F I G **K** 520

CO/17 1547 CTC AAG ATG ATG AAT GAC TCA ATG GCT AAG AAA ACC AGT GGA AAT GCT TTC ATT GGG AAG 1606

||| ||| ||| ||| ||| ||| ||| ||| ||| ||| ||| ||| ||| ||| ||| ||| ||| || ||| |||

PK/13 1501 CTC AAG ATG ATG AAT GAC TCA ATG GCT AAG AAA ACC AGT GGA AAT GCT TTC GTT GGG AAG 1560

K M F Q I S D K N K T N P **II** E I P I K Q 540

CO/17 1607 AAA ATG TTT CAA ATA TCA GAC AAA AAC AAA ACC AAT CCC ATT GAA ATT CCA ATT AAG CAG 1666

||| ||| ||| ||| ||| ||| ||| ||| ||| ||| ||| ||| ||| ||| ||| ||| ||| ||| ||| |||

PK/13 1561 AAA ATG TTT CAA ATA TCA GAC AAA AAC AAA ACC AAT CCC ATT GAA ATT CCA ATT AAG CAG 1620

T I P N F F F G R D T A E D Y D D L D Y 560

CO/17 1667 ACC ATC CCC AAT TTC TTC TTT GGG AGG GAC ACA GCA GAG GAT TAT GAT GAC CTC GAT TAT 1726

||| ||| ||| ||| ||| ||| ||| ||| ||| ||| ||| ||| ||| ||| ||| ||| ||| ||| ||| |||

PK/13 1621 ACC ATC CCC AAT TTC TTC TTT GGG AGG GAC ACA GCA GAG GAT TAT GAT GAC CTC GAT TAT 1680

CO/17 1727 TAA 1729

|||

PK/13 1681 TAA 1683

Protein numbering is shown on the right side for the full length NP ORF.

Residues highlighted in **bold** were identified in Chen and Holmes (2008) Table 2 as positively selected amino acid positions, 0/2 NP were subject to mutation in this analysis.

Residues highlighted in **bold** were identified in Yang et al., (2012) Table 2 as amino acid substitutions in multiple genotypes, 0/1 NP were subject to mutation in this analysis.

Residues highlighted in **bold** were identified in Virk et al., (2020) supplementary information as truck amino acid substitutions on phylogenetic trees, 4/8 NP were subject to mutation in this analysis. Amino acid positions identified as positively selected sites are marked with an asterisk *****

**NA**

**NB**  M N N A T F N Y T **9**

NA 1 M L P S T I Q 7

CO/17 1 ATCTTCTC--AAAACTGAAGCAAATAGGCCAAAA ATG AAC AATGCTACCTTCAACTATAC 58

|||||||| |||||||| ||||||||||||||| ||| ||| ||||||||||||||||||||

PK/13 1 ATCTTCTCAAAAAACTGAGGCAAATAGGCCAAAA ATG AAC AATGCTACCTTCAACTATAC 60

N V N **P** I S H I R G S I I I T I C V S F **29**

T L T L **F** L T S G G V***** L L S L Y V S A S 27

CO/17 59 AAACGTTAACCCTATTTCTCACATCAGGGGGAGTATTATTATCACTATATGTGTCAGCTT 118

||||||||||| |||||||||||||||||||||| |||||||||||||||||||||||||

PK/13 61 AAACGTTAACCTTATTTCTCACATCAGGGGGAGTGTTATTATCACTATATGTGTCAGCTT 120

I I I L T I L G Y I A K I L T **N** R N N C **49**

L S Y L L Y S***** D I L L K F S **PP** T E **I*** T A 47

CO/17 119 CATTATCATACTTACTATACTCGGATATATTGCTAAAATTCTCACCAACAGAAATAACTG 178

|||| |||||||||||||| |||||||||||||||||||| |||| ||||||| ||||||

PK/13 121 CATTGTCATACTTACTATATTCGGATATATTGCTAAAATTTTCACGAACAGAAGTAACTG 180

T N N A I G L C K R I K C S G C E **P** F C **69**

***** P **T** M P L D C A N A S N V Q A V N***** **R** S A 67

CO/17 179 CACCAACAATGCCATTGGATTGTGCAAACGCATCAAATGTTCAGGCTGTGAACCGTTCTG 238

|||||| |||||||||||||||||||||||||||||||||||||||||||||||||||||

PK/13 181 CACCAATAATGCCATTGGATTGTGCAAACGCATCAAATGTTCAGGCTGTGAACCGTTCTG 240

N K R G D T S S P R T G **V** D **I** P A F I L **89**

**T** K G V **T** **L*** L L P E P E W T Y P***** R L S C 87

CO/17 239 CAACAAAAGGGGTGACACTTCTTCTCCCAGAACCGGAGTGGACATACCCGCGTTTATCTT 298

|||||||||||||||||| |||||||||||||||||||||||| ||||| ||||||||||

PK/13 241 CAACAAAAGGGGTGACACCTCTTCTCCCAGAACCGGAGTGGACGTACCCTCGTTTATCTT 300

P G L N L S E S T P N *

P G***** S T F Q K A L L I S P H R F***** G E **TT*** K 107

CO/17 299 GCCCGGGCTCAACCTTTCAGAAAGCACTCCTAATTAGC CCT CAT AGA TTC GGA GAA ACC A 358

|||||||||||||||||||||||||||||||||||||| || ||| ||| ||| ||| ||| ||| |

PK/13 301 GCCCGGGCTCAACCTTTCAGAAAGCACTCCTAATTAGC CCC CAT AGA TTC GGA GAA ACC A 360

G N S A P L I I R E P F **V** A C G P **NN** E C 127

CO/17 359 AA GGA AAC TCA GCT CCC TTG ATA ATA AGG GAA CCT TTT GTT GCT TGT GGA CCA AAT GAA T 418

|| ||| ||| ||| ||| ||| ||| ||| ||| ||| ||| ||| ||| || ||| ||| ||| ||| || ||| |

PK/13 361 AA GGA AAC TCA GCT CCC TTG ATA ATA AGG GAA CCT TTT ATT GCT TGT GGA CCA AAG GAA T 420

K H F A L T H Y A A Q P G G Y Y N G T R 147

CO/17 419 GC AAA CAC TTT GCT TTA ACC CAT TAT GCA GCC CAA CCA GGG GGA TAC TAC AAT GGA ACA A 478

|| ||| || ||| ||| || ||| ||| ||| ||| || ||| ||| ||| ||| ||| ||| ||| ||| ||| |

PK/13 421 GC AAA CAT TTT GCT CTA ACC CAT TAT GCA GCT CAA CCA GGG GGA TAC TAC AAT GGA ACA A 480

G D R N K L R H L I S V K L G***** K I P T V 167

CO/17 479 GA GGA GAC AGA AAC AAG CTG AGG CAT CTA ATT TCA GTC AAA TTG GGC AAA ATC CCA ACA G 538

|| | | ||| ||| ||| ||| ||| ||| ||| ||| ||| ||| ||| ||| ||| ||| ||| ||| ||| ||| |

PK/13 481 GA GAA GAC AGA AAC AAG CTG AGG CAT CTA ATT TCA GTC AAA TTG GGC AAA ATC CCA ACA G 540

E N S **I** F***** H M A A W S G S A***** C H D G **KK*** E 187

CO/17 539 TA GAG AAC TCC ATT TTC CAC ATG GCA GCA TGG AGC GGG TCC GCG TGC CAT GAT GGT AAG G 598

|| || ||| || ||| ||| ||| ||| ||| || ||| ||| ||| ||| || ||| ||| ||| ||| | |

PK/13 541 TA GAA AAC TCT ATT TTC CAC ATG GCA GCT TGG AGC GGG TCC GCA TGC CAT GAT GGT AGA G 600

W T Y I***** G V D G P***** D N N A L L K V K Y G 207

CO/17 599 AA TGG ACA TAT ATC GGA GTT GAT GGC CCT GAC AAT AAT GCA TTG CTC AAA GTA AAA TAT G 658

|| ||| || || ||| ||| ||| ||| ||| || ||| | | ||| ||| ||| ||| ||| || ||| ||| |

PK/13 601 AA TGG ACT TAC ATC GGA GTT GAT GGC CCA GAC AGT AAT GCA TTG CTC AAA ATA AAA TAT G 660

E A Y T D T Y H S Y A N **N** I L R T Q E S 227

CO/17 659 GA GAA GCA TAT ACT GAC ACA TAC CAT TCC TAT GCA AAC AAC ATC CTA AGA ACA CAA GAA A 718

|| ||| ||| ||| ||| ||| ||| ||| ||| ||| ||| ||| || ||| ||| ||| || ||| ||| ||| |

PK/13 661 GA GAA GCA TAT ACT GAC ACA TAC CAT TCC TAT GCA AAA AAC ATC CTA AGG ACA CAA GAA A 720

A C N C I G G N C Y L M I T D G S A S G 247

CO/17 719 GT GCC TGC AAT TGC ATC GGG GGA AAT TGT TAT CTA ATG ATA ACT GAT GGC TCA GCT TCA G 778

|| ||| ||| ||| ||| ||| ||| ||| || ||| ||| || ||| ||| ||| ||| ||| || ||| ||| |

PK/13 721 GT GCC TGC AAT TGC ATC GGG GGA GAT TGT TAT CTG ATG ATA ACT GAT GGC CCA GCT TCA G 780

**VV** S E C R F L K I R E G R I I K E I F P 267

CO/17 779 GT GTT AGT GAA TGC AGA TTT CTT AAG ATT CGA GAG GGC CGA ATA ATA AAA GAA ATA TTT C 838

| || ||| ||| ||| ||| || ||| ||| ||| ||| ||| ||| ||| ||| ||| ||| ||| ||| ||| |

PK/13 781 GG ATT AGT GAA TGC AGA TTC CTT AAG ATT CGA GAG GGC CGA ATA ATA AAA GAA ATA TTT C 840

T G R V***** K H T E E C T C G F A***** S N K T I 287

CO/17 839 CA ACA GGA AGA GTA AAA CAC ACT GAG GAA TGC ACA TGC GGA TTT GCC AGC AAT AAA ACC A 898

|| ||| ||| ||| ||| ||| || ||| ||| ||| ||| ||| ||| ||| ||| ||| ||| || ||| ||| |

PK/13 841 CA ACA GGA AGA GTA AAA CAT ACT GAG GAA TGC ACA TGC GGA TTT GCC AGC AAC AAA ACC A 900

E C A C R D N **RR** Y T A K R P F V K L N V 307

CO/17 899 TA GAA TGT GCC TGT AGA GAC AAC AGG TAC ACA GCA AAA AGA CCT TTT GTC AAA TTA AAC G 958

|| ||| ||| ||| ||| ||| || ||| || ||| ||| ||| ||| ||| || ||| ||| ||| ||| || |

PK/13 901 TA GAA TGT GCC TGT AGA GAT AAC AGT TAC ACA GCA AAA AGA CCC TTT GTC AAA TTA AAT G 960

E***** T D T A E I R L M C T **D** T Y L D T***** P R 327

CO/17 959 TG GAG ACT GAT ACA GCA GAA ATA AGG TTG ATG TGC ACA GAT ACC TAT TTG GAC ACC CCC A 1018

|| ||| ||| ||| ||| || ||| ||| || ||| ||| ||| ||| | || ||| ||| ||| ||| ||| |

PK/13 961 TG GAG ACT GAT ACA GCG GAA ATA AGA TTG ATG TGC ACA AAG ACT TAT TTG GAC ACC CCC A 1020

P **N** D G S I T G P C E S **DD** G **D** **K** G **S** G G 347

CO/17 1019 GA CCA AAT GAT GGA AGC ATA ACA GGC CCT TGT GAA TCT GAT GGG GAC AAA GGG AGT GGA G 1078

|| ||| ||| ||| ||| ||| ||| ||| || ||| || ||| ||| ||| ||| ||| || ||| ||| ||| |

PK/13 1021 GA CCA AAT GAT GGA AGC ATA ACA GGG CCT TGC GAA TCT GAT GGG GAC GAA GGG AGT GGA G 1080

I K G G F***** V H Q R M **K*** S K I G R W Y S R 367

CO/17 1079 GC ATC AAG GGA GGA TTT GTT CAT CAA AGA ATG AAA TCC AAG ATT GGA AGG TGG TAC TCT C 1138

|| ||| ||| ||| ||| ||| ||| || ||| ||| ||| | ||| ||| ||| ||| ||| ||| ||| ||| |

PK/13 1081 GC ATC AAG GGA GGA TTT GTT CAC CAA AGA ATG GCA TCC AAG ATT GGA AGG TGG TAC TCT C 1140

T***** **M** S **Q** T **EE** R M G M **G** L Y V K Y **G** G D P 387

CO/17 1139 GA ACG ATG TCT CAA ACT GAA AGG ATG GGG ATG GGA CTG TAT GTC AAG TAT GGT GGA GAC C 1198

| ||| ||| ||| || ||| || || ||| ||| ||| ||| ||| ||| || ||| ||| | | ||| ||| |

PK/13 1141 GG ACG ATG TCT AAA ACT AAA AGA ATG GGG ATG GGA CTG TAT GTA AAG TAT GAT GGA GAC C 1200

W A D S D***** A L **A**** **F** S G V M **V** **S** M K E P G 407

CO/17 1199 CA TGG GCT GAC AGT GAT GCC CTA GCT TTT AGT GGA GTA ATG GTT TCA ATG AAA GAA CCT G 1258

|| ||| || ||| ||| || ||| || ||| || ||| ||| ||| ||| ||| ||| ||| || ||| ||| |

PK/13 1201 CA TGG ACT GAC AGT GAA GCC CTT GCT CTT AGT GGA GTA ATG GTT TCA ATG GAA GAA CCT G 1260

W Y***** S F G***** F E I K D K K C***** D V P C I G I 427

CO/17 1259 GT TGG TAT TCC TTT GGC TTC GAA ATA AAA GAT AAG AAA TGC GAT GTC CCC TGT ATT GGG A 1318

|| ||| ||| ||| ||| ||| ||| ||| ||| ||| ||| ||| ||| || ||| ||| ||| ||| ||| ||| |

PK/13 1261 GT TGG TAT TCC TTT GGC TTC GAA ATA AAA GAT AAG AAA TGT GAT GTC CCC TGT ATT GGG A 1320

E M V***** H D G G***** K E***** T W H S A***** A T A I Y C 447

CO/17 1319 TA GAG ATG GTA CAT GAT GGT GGA AAA GAG ACT TGG CAC TCA GCA GCA ACA GCC ATT TAC T 1378

|| || ||| ||| ||| ||| ||| || ||| | ||| ||| ||| ||| || ||| ||| ||| ||| ||| |

PK/13 1321 TA GAA ATG GTA CAT GAT GGT GGG AAA ACG ACT TGG CAC TCA GCG GCA ACA GCC ATT TAC T 1380

L M G S G Q L L***** W D T V T G***** V **DD** M **AA** L * 466

CO/17 1379 GT TTA ATG GGC TCA GGA CAG CTG CTG TGG GAC ACT GTC ACA GGT GTT GAC ATG GCT CTG T 1438

|| ||| ||| ||| ||| ||| || ||| ||| ||| ||| ||| ||| ||| ||| ||| | ||| || ||| |

PK/13 1381 GT TTA ATG GGC TCA GGA CAA CTG CTG TGG GAC ACT GTC ACA GGT GTT AAT ATG ACT CTG T 1440

CO/17 1439 AATGGAGGAGTGGTTAAGTCTGTTCTAAACCCTTTGTTCCTATTTTGTTTGAACAATTGT 1498

||||||||| ||||| ||||||||||||||||||||||||||||||||||||||||||||

PK/13 1441 AATGGAGGAATGGTTGAGTCTGTTCTAAACCCTTTGTTCCTATTTTGTTTGAACAATTGT 1500

CO/17 1499 CCTTACTAGACTTAATTGTTTCTGAAAAAT 1528

||||||| |||| ||||||||||||||||

PK/13 1501 CCTTACTGAACTTGATTGTTTCTGAAAAAT 1530

Protein numbering is shown on the right side for the full length NA and NB (**bold**) ORFs.

Residues highlighted in **bold** were identified in Chen and Holmes (2008) Table 2 as positively selected amino acid positions, 2/3 NA were subject to mutation in this analysis.

Residues highlighted in **bold** were identified in Yang et al., (2012) Table 2 as amino acid substitutions in multiple genotypes, 1/5 NB and 7/9 NA were subject to mutation in this analysis.

Amino acid positions identified as positively selected sites in Yoshihara et al., (2020) are marked with an asterisk *****, 0/2 NA were subject to mutation in this analysis. Amino acid positions identified as negatively selected sites are marked with an asterisk *****, 15/25 NA were subject to mutation in this analysis.

Residues highlighted in **bold** were identified in Virk et al., (2020) as truck amino acid substitutions on phylogenetic trees, 16/32 NA were subject to mutation in this analysis. Amino acid positions identified as positively selected sites are marked with an asterisk *****

**M**

1 M S L F G D T I A Y L L S L T E D G E G 20

CO/17 8 ATG TCG CTG TTT GGA GAC ACA ATT GCC TAC TTG CTT TCA TTG ACA GAA GAT GGA GAA GGC 67

||| ||| ||| ||| ||| ||| ||| ||| ||| ||| || ||| ||| ||| ||| ||| ||| ||| ||| |||

PK/13 1 ATG TCG CTG TTT GGA GAC ACA ATT GCC TAC CTG CTT TCA TTG ACA GAA GAT GGA GAA GGC 60

K A E L A E K L H C W F G G K E F D L D 40

CO/17 68 AAA GCA GAA CTA GCA GAA AAG TTA CAC TGT TGG TTT GGT GGG AAA GAA TTT GAC CTA GAC 127

||| ||| ||| ||| ||| ||| || ||| ||| ||| ||| || ||| ||| ||| ||| ||| ||| ||| |||

PK/13 61 AAA GCA GAA CTA GCA GAA AAA TTA CAC TGT TGG TTC GGT GGG AAA GAA TTT GAC CTA GAC 120

S A L E W I K N K R C L T D I Q K A L I 60

CO/17 128 TCA GCC TTG GAA TGG ATA AAA AAC AAA AGA TGC TTA ACT GAT ATA CAA AAA GCA CTA ATT 187

|| ||| ||| ||| ||| ||| ||| ||| ||| ||| ||| ||| ||| ||| ||| || ||| ||| ||| |||

PK/13 121 TCT GCC TTG GAA TGG ATA AAA AAC AAA AGA TGC TTA ACT GAT ATA CA**A** AAA GCA CTA ATT 180

G A S I C F L K P K D Q E R K R R F I T 80

CO/17 188 GGT GCC TCT ATA TGC TTT TTA AAA CCC AAA GAC CAG GAA AGA AAA AGA AGA TTC ATC ACA 247

||| ||| ||| || ||| ||| ||| ||| ||| ||| ||| ||| ||| ||| ||| ||| ||| ||| ||| |||

PK/13 181 GGT GCC TCT AT**A** TGC TTT TTA AAA CCC AAA GAC CAG GAA AGA AAA AGA AGA TTC ATC ACA 240

E P L S G M G T T A T K K K G L I L A E 100

CO/17 248 GAG CCC TTA TCA GGA ATG GGA ACA ACA GCA ACA AAA AAG AAA GGC CTG ATT CTG GCT GAG 307

||| ||| ||| || ||| ||| ||| ||| ||| ||| ||| ||| ||| || ||| ||| ||| ||| ||| |||

PK/13 241 GAG CCC TTA TC**A** GGA ATG GGA ACA ACA GCA ACA AAA AAG AA**A** GGC CTG ATT CTG GCT GAG 300

R K M R **R** C V S F H E A F E I A E G H E 120

CO/17 308 AGA AAA ATG AGA AGA TGT GTG AGC TTT CAT GAA GCA TTT GAA ATA GCA GAA GGC CAT GAA 367

||| ||| ||| ||| | | ||| ||| ||| ||| ||| ||| ||| ||| ||| ||| ||| ||| ||| ||| |||

PK/13 301 AGA AAA ATG AGA A**G**A TGT GTG AGC TTT CAT GAA GCA TTT GAA ATA GCA GAA GGC CAT GAA 360

S S A L L Y C L M V M Y L N P G N Y S M 140

CO/17 368 AGC TCA GCG CTA CTA TAC TGT CTC ATG GTC ATG TAC CTG AAT CCT GGA AAT TAT TCA ATG 427

||| ||| ||| ||| ||| || ||| ||| ||| ||| ||| ||| ||| ||| ||| ||| ||| ||| ||| |||

PK/13 361 AGC TCA GCG CTA CTA TA**C** TGT CTC ATG GTC ATG TAC CTG AAT CCT GGA AAT TAT TCA ATG 420

Q V K L G T L C A L C E K Q A S H S H R 160

CO/17 428 CAA GTA AAA CTA GGA ACG CTC TGT GCT TTA TGC GAG AAA CAA GCA TCA CAT TCA CAC AGG 487

||| ||| ||| ||| ||| ||| ||| ||| ||| || ||| || ||| ||| || ||| ||| ||| ||| ||

PK/13 421 CAA GTA AAA CTA GGA ACG CTC TGT GCT TT**A** TGC GA**G** AAA CAA GCT TCA CAT TCA CAC AG**G** 480

A H S R A A R S S V P G V R R E M Q M V 180

CO/17 488 GCT CAT AGC AGA GCA GCG AGA TCT TCA GTG CCT GGA GTG AGA CGA GAA ATG CAG ATG GTC 547

||| ||| ||| ||| ||| ||| ||| ||| ||| ||| || ||| ||| ||| || ||| ||| ||| ||| |||

PK/13 481 GCT CAT AGC AGA GCA GCG AGA TCT TCA GTG CC**T** GGA GTG AGA CG**A** GAA ATG CAG ATG GTC 540

S A M N T A K T M N G M G K G E D V Q K 200

CO/17 548 TCA GCT ATG AAC ACA GCA AAA ACA ATG AAT GGA ATG GGA AAA GGA GAA GAT GTC CAA AAG 607

||| ||| ||| ||| ||| ||| ||| ||| ||| ||| ||| ||| ||| ||| ||| || || ||| ||| ||

PK/13 541 TCA GCT ATG AAC ACA GCA AAA ACA ATG AAT GGA ATG GGA AAA GGA GAG GAC GTC CAA AA**G** 600

L A E E L Q S N I G V L R S L G A S Q K 220

CO/17 608 CTG GCA GAA GAG TTG CAA AGC AAC ATC GGA GTG CTG AGA TCT CTT GGG GCA AGT CAA AAG 667

||| ||| ||| || || ||| ||| ||| || ||| || || ||| || ||| ||| ||| ||| || |||

PK/13 601 CTG GCA GAA GA**G** **T**TG CAA AGC AAC ATT GGA GT**G** **C**TG AGA TC**T** CTT GGG GCA AGT CA**A** AAG 660

N G E G I A K D V M E V L K Q S S M G N 240

CO/17 668 AAT GGA GAA GGG ATT GCA AAG GAT GTA ATG GAA GTG CTA AAG CAG AGC TCC ATG GGA AAT 727

||| || ||| || ||| ||| ||| ||| ||| ||| ||| ||| ||| ||| ||| ||| || ||| ||| |||

PK/13 661 AAT GGG GAA GG**G** ATT GCA AAG GAT GTA ATG GAA GTG CTA AAG CAG AGC TC**C** ATG GGA AAT 720

S A L V K K Y L * M L E P F Q I L T I C S **12**

CO/17 728 TCA GCT CTT GTG AAG AAA TAT CTA TAATG CTC GAA CCA TTT CAG ATT CTT ACA ATT TGT T 787

||| ||| ||| ||| ||| ||| || ||| ||||| | ||| ||| || ||| ||| ||| || ||| ||| |

PK/13 721 TCA GCT CTT GTG AAG AAA TA**T** CTA TAATG **C**T**C** GAA CCA TT**T** CAG **G**TT CTT **A**CA ATT TGT T 780

F I L S A L H F **M** A W T I G H L N Q I K **32**

CO/17 788 CT TTT ATC TTA TCA GCT CTC CAT TTC ATG GCT TGG ACA ATA GGG CAT TTG AAT CAA ATA A 847

|| ||| || ||| ||| ||| ||| ||| ||| ||| ||| ||| ||| ||| || ||| || ||| ||| ||| |

PK/13 781 CT TTT AT**C** TTA TCA GCT CTC CAT TTC ATG GCT TGG ACA ATA GG**G** CAT TT**G** AAT CAA ATA A 840

R G I N M K I R I K G P N K E T I T R E **52**

CO/17 848 AA AGA GGA ATA AAC ATG AAA ATA CGA ATA AAA GGT CCA AAC AAA GAG ACA ATA ACC AGA G 907

|| ||| ||| || ||| ||| ||| ||| || ||| || || ||| || ||| ||| ||| ||| | | ||| |

PK/13 841 AA AGA GGA **A**TA AAC ATG AAA ATA **C**GA ATA AA**A** GG**T** CCA AA**C** AAA GAG ACA ATA AAC AGA G 900

V S I L R H **S** Y Q K E I Q A K E T M K E **72**

CO/17 908 AG GTA TCA ATT TTG AGA CAC AGT TAC CAA AAA GAA ATC CAG GCC AAA GAA ACA ATG AAG G 967

|| ||| ||| ||| ||| ||| ||| ||| ||| ||| ||| ||| || ||| || || ||| || ||| ||| |

PK/13 901 AG GTA TCA ATT TTG AGA CAC AGT TAC CAA AAA GAA AT**C** CAG GC**C** AA**A** GAA **A**CA ATG AAG G 960

V L S D N M E V L N D H I I I E G L S A **92**

CO/17 968 AA GTG CTC TCT GAC AAC ATG GAG GTA TTG AAT GAC CAC ATA ATA ATT GAG GGG CTT TCT G 1027

|| || ||| ||| ||| ||| ||| || ||| ||| | | ||| ||| ||| || ||| ||| ||| ||| ||| |

PK/13 961 AA GT**G** CTC TCT GAC AAC ATG GA**G** GTA TTG A**A**T GAC CAC ATA **A**TA ATT GAG GGG CTT TCT G 1020

E E I I K M G E T V L E I E E **L** H *

CO/17 1028 CC GAA GAG ATA ATA AAA ATG GGT GAA ACA GTT TTG GAG ATA GAA GAA TTG CAT TAA ATTC 1087

| ||| ||| ||| ||| ||| ||| ||| ||| ||| ||| ||| ||| || ||| ||| | | ||| ||| ||||

PK/13 1021 CT GAA GAG ATA ATA AAA ATG GGT GAA ACA GTT TTG GAG GTA GAA GAA TCG CAT TAA ATTC 1080

CO/17 1088 AA-TTTTACTGTATTTCTTACTATGCATTTAAGCAAATTGTAATCAATGTCAGCAAATAA 1146

|| |||||||||||||| ||||||||||||||||||||||||||||||||||||||||||

PK/13 1081 AATTTTTACTGTATTTCCTACTATGCATTTAAGCAAATTGTAATCAATGTCAGCAAATAA 1140

Protein numbering is shown on the right side for the full length M1 and M2 (**bold**) ORFs.

Residues highlighted in **bold** were identified in Yang et al., (2012) Table 2 as amino acid substitutions in multiple genotypes, 1/1 M1 and 1/3 BM2 were subject to mutation in this analysis.

**NS**

**NS2** M A N N N M T T T Q I E **12**

NS1 1 M A N N N M T T T Q I E V G P G A T N A T I N 23

Qu 31 atg gcg aac AAC AAC ATG ACC ACA ACA CAA ATT GAG GTG GGT CCG GGA GCA ACC AAT GCC ACC ATA AAC 96

||| ||| ||| || ||| ||| ||| ||| ||| ||| ||| ||| ||| ||| ||| ||| ||| ||| ||| ||| ||| |||

Sb 1 atg gcg AAC AA**C** ATG ACC ACA ACA CAA ATT GAG GTG GGT CCG GGA GCA ACC AAT GCC ACC ATA AAC 66

F E A G I L E C Y E R L S W Q R A L D Y 43

CO/17 97 TTT GAA GCA GGA ATT CTG GAG TGC TAT GAA AGG CTT TCA TGG CAA AGA GCC CTT GAC TAC 156

||| ||| ||| ||| ||| ||| ||| ||| ||| ||| || ||| ||| ||| ||| || ||| ||| ||| |||

PK/13 67 TTT GAA GCA GGA ATT CTG GAG TGC TAT GAA AG**G** CTT TCA TGG CAA AG**A** GCC CTT GAC TAC 126

P G Q D R L N R L K R K L E S R I K T H 63

CO/17 157 CCC GGT CAA GAC CGC CTA AAC AGA CTA AAA AGA AAA TTA GAG TCA AGA ATA AAG ACT CAC 216

|| ||| ||| ||| || ||| ||| ||| ||| || || ||| ||| ||| ||| ||| ||| ||| ||| |||

PK/13 127 CC**C** GGT CAA GAC CG**C** CTA AAC AGA CTA AA**A** AG**A** AAA TTA GAG TCA AGA ATA AAG ACT CAC 186

N K S E P E S K R M S L E E R K A I G V 83

CO/17 217 AAC AAA AGT GAG CCT GAA AGT AAA AGG ATG TCC CTT GAA GAG AGA AAA GCA ATT GGA GTA 276

||| ||| ||| ||| ||| ||| ||| ||| ||| ||| || ||| ||| ||| ||| ||| ||| ||| ||| |||

PK/13 187 AAC AAA AGT GAG CCT GAA AGT AAA AGG ATG TC**C** CTT GAA GAG AGA AAA GCA ATT GGA GTA 246

K M M **K** V L L F M N P S A G I E G F E P 103

CO/17 277 AAA ATG ATG AAA GTA CTC CTA TTT ATG AAT CCG TCT GCT GGA ATT GAA GGG TTT GAG CCA 336

||| ||| ||| ||| ||| || ||| ||| ||| ||| ||| ||| ||| ||| ||| ||| ||| ||| ||| |||

PK/13 247 AAA ATG ATG AAA GTA CT**C** CTA TTT ATG AAT CCG TCT GCT GGA ATT GAA GGG TTT GAG CCA 306

Y C **M** **N** S S S **NN** S N C **TTT** K Y **N** W **TT** **D** Y P***** 123

CO/17 337 TAC TGT ATG AAC AGT TCC TCA AAT AGC AAC TGT ACG AAA TAC AAT TGG ACC GAT TAC CCT 396

||| ||| ||| || ||| ||| ||| || ||| ||| ||| || ||| ||| | | ||| | ||| ||| |||

PK/13 307 TAC TGT ATG AA**C** AGT TCC TCA AA**T** AGC AAC TGT **A**CG AAA TAC A**A**T TGG A**CC** GAT TAC CCT 366

S T P **E** R C L D D I E E E P **E** D V D G P 143

CO/17 397 TCA ACA CCA GAG AGG TGC CTT GAT GAC ATA GAG GAA GAA CCA GAG GAT GTT GAT GGC CCA 456

||| || ||| | | ||| ||| ||| ||| ||| ||| || ||| ||| ||| || ||| ||| ||| ||| |||

PK/13 367 TCA AC**A** CCA G**A**G AGG TGC CTT GAT GAC ATA GA**G** GAA GAA CCA GA**G** GAT GTT GAT GGC CCA 426

T E I V L R D M N N **K** D A R Q K I K E E 163

CO/17 457 ACT GAA ATA GTA TTA AGG GAC ATG AAC AAC AAA GAT GCA AGG CAA AAG ATA AAG GAG GAA 516

||| ||| ||| ||| ||| ||| ||| ||| ||| ||| ||| ||| ||| ||| ||| ||| ||| ||| ||| |||

PK/13 427 ACT GAA ATA GTA TTA AGG GAC ATG AAC AAC AAA GAT GCA AGG CAA AAG ATA AAG GAG GAA 486

V N T Q K E G K F R L T I **K** R D **MM** R N V 183

CO/17 517 GTA AAC ACT CAG AAA GAA GGG AAG TTC CGT TTG ACA ATA AAA AGG GAT ATG CGT AAT GTA 576

||| ||| ||| ||| ||| ||| ||| ||| ||| ||| ||| ||| ||| ||| ||| ||| ||| ||| ||| |||

PK/13 487 GTA AAC ACT CAG AAA GAA GGG AAG TTC CGT TTG ACA ATA AAA AGG GAT ATG CGT AAT GTA 546

L S L R V L V N G T F **L** K H P N***** G Y K S 203

CO/17 577 TTG TCC TTG AGA GTG TTG GTA AAT GGA ACA TTC CTC AAA CAC CCC AAT GGA TAC AAG TCC 636

||| ||| || ||| ||| || ||| || ||| ||| ||| ||| ||| ||| ||| ||| ||| ||| ||| |||

PK/13 547 TTG TCC **T**TG AGA GTG TT**G** GTA AA**T** GGA ACA TTC CTC AAA CAC CCC AAT GGA TAC AAG TCC 606

L S***** T L H R L N A Y D Q S G R L V A K L 223

CO/17 637 TTA TCA ACT CTG CAT AGA TTG AAT GCA TAT GAC CAG AGT GGA AGG CTT GTT GCT AAA CTT 696

||| ||| ||| ||| ||| ||| || ||| ||| ||| ||| ||| ||| ||| ||| ||| ||| ||| ||| |||

PK/13 607 TTA TCA ACT CTG CAT AGA CTG AAT GCA TAT GAC CAG AGT GGA AGG CTT GTT GCT AAA CTT 666

W R M K K M A I G S S T H **25**

V A T D D L T V E D E E D G H R I **L** N S 243

CO/17 697 GTT GCC ACT GAT GAT CTT ACA GTGGAGGATGAAGAAGATGGCCATCGGATCCTCAACTCA 756

||| || ||| ||| ||| ||| ||| ||||||||||||||||||||||||||||||||||| |||

PK/13 667 GTT GC**C** ACT GAT GAT CTT ACA GTGGAGGATGAAGAAGATGGCCATCGGATCCTCAA**C**TCA 726

S S S V S M K D I Q S Q F E Q L K L R W **45**

L F E R L N E G H S***** K P I R A A E T A V 263

CO/17 757 CTCTTCGAGCGTCTCAATGAAGGACATTCAAAGCCAATTCGAGCAGCTGAAACTGCGGTG 816

|||||||||||||| |||||||||||||||||||||||||||||||||||||||||||||

PK/13 727 CTCTTCGAGCGTCTTAATGAAGGACATTCAAAGCCAATTCGAGCAGCTGAAACTGCGGTG 786

E S Y P N L V K S T D Y H Q K R E T I R **65**

G V L S Q F G Q E H R L S P E E G D N *

CO/17 817 GGAGTCTTATCCCAATTTGGTCAAGAGCACCGATTATCACCAGAAGAGGGAGACAATTAG 876

||||||||||||||||||||||||||||||||||||||||||||||||||||||||||||

PK/13 787 GGAGTCTTATCCCAATTTGGTCAAGAGCACCGATTATCACCAGAAGAGGGAGACAATTAG 846

L V T E E L Y L L S K R I D D N I L F H **85**

CO/17 877 A TTG GTC ACG GAA GAA CTT TAT CTT TTA AGT AAA AGA ATT GAT GAT AAC ATA CTA TTC CA 936

| || ||| ||| ||| ||| ||| ||| ||| ||| ||| ||| ||| ||| ||| ||| ||| ||| | ||| ||

PK/13 847 A **T**TG GTC ACG GAA GAA CTT TAT CTT TTA AGT AAA AGA ATT GAT GAT AAC ATA **C**T**A** TTC CA 906

K T **V** I A N S S I I A D M V V S L S L L **105**

CO/17 937 C AAA ACA GTG ATA GCT AAC AGC TCC ATA ATA GCT GAC ATG GTT GTA TCA TTA TCA TTA TT 996

| ||| ||| || ||| ||| ||| ||| ||| ||| ||| ||| ||| ||| ||| ||| ||| ||| ||| ||| ||

PK/13 907 C AAA ACA GT**G** ATA GCT AAC AGC TCC ATA ATA GCT GAC ATG GTT GTA TCA TTA TCA TTA TT 966

E T L Y E M K D V V E V Y S R Q C L * **123**

CO/17 997 A GAA ACA TTG TAT GAA ATG AAG GAT GTG GTT GAA GTG TAC AGC AGG CAG TGC TTG TGA AT 1056

| ||| ||| ||| ||| ||| ||| ||| ||| ||| ||| ||| ||| ||| ||| ||| ||| ||| ||| ||| ||

PK/13 967 A GAA ACA TTG TAT GAA ATG AAG GAT GTG GTT GAA GTG TAC AGC AGG CAG TGC TTG TGA AT 1026

CO/17 1057 TTAAAATAAA 1066

||||||||||

PK/13 1027 TTAAAATAAA 1036

Protein numbering is shown on the right side for the NS1 and NS2 (**bold**) ORFs.

Residues highlighted in **bold** were identified in Chen and Holmes (2008) Table 2 as positively selected amino acid positions, 1/2 NS1 were subject to mutation in this analysis.

Residues highlighted in **bold** were identified in Yang et al., (2012) Table 2 as amino acid substitutions in multiple genotypes, 3/4 NS1 and 1/1 NS2 were subject to mutation in this analysis.

Residues highlighted in **bold** were identified in Virk et al., (2020) supplementary information as truck amino acid substitutions on phylogenetic trees, 7/14 NS were subject to mutation in this analysis (different Victoria & Yamagata NS1 lengths). Amino acid positions identified as positively selected sites are marked with an asterisk *****
