# Supplementary material for: Insights from the comparison of genomic variants from two influenza B viruses grown in the presence of human antibodies in cell culture
Source: PLoS One. 2020 Sep 14;15(9):e0239015. doi: 10.1371/journal.pone.0239015 (PMC7489522; doi:10.1371/journal.pone.0239015)

**S1 Figure. Number of Loci with Variation >5% in Escape Viruses.**

The number of loci with variants present at levels greater than 5% in the NGS data are graphed for each escape virus. The viruses identified with CO are derived from the Victoria-lineage virus B/Colorado/06/2017 and the viruses with PK are derived from the Yamagata-lineage virus B/Phuket/3073/2013. Blue bars indicate loci where a variant increased in frequency compared to that in the virus grown without antibodies, orange bars indicate loci where a variant decreased in frequency compared to that in the virus grown without antibodies.


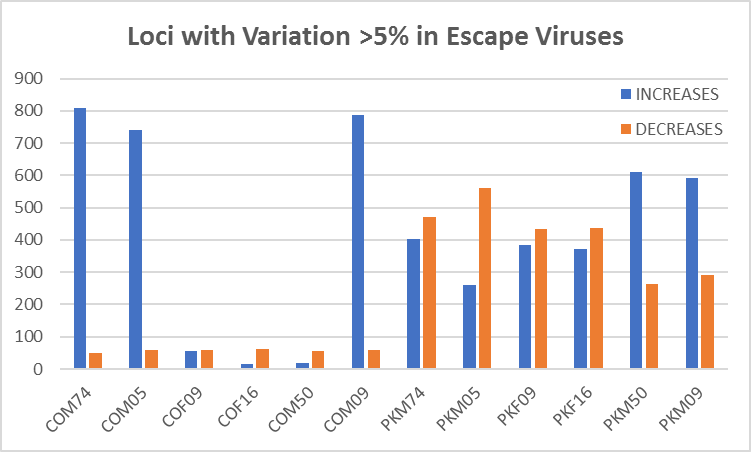

Supplement: S1 Fig — The number of loci with variants present at levels greater than 5% in the NGS data are graphed for each escape virus. The viruses identified with CO are derived from the Victoria-lineage virus B/Colorado/06/2017 and the viruses with PK are derived from the Yamagata-lineage virus B/Phuket/3073/2013. Blue bars indicate loci where a variant increased in frequency compared to that in the virus grown without antibodies, orange bars indicate loci where a variant decreased in frequency compared to that in the virus grown without antibodies. (DOCX) [file pone.0239015.s010.docx]
